# Study on the preparation of compound mold enhanced *Xiaoqu* and its effect on the yield and flavor of *Qingxiangxing baijiu*

**DOI:** 10.1016/j.fochx.2025.102721

**Published:** 2025-07-03

**Authors:** Tongwei Guan, Xinrui Yin, Yuhang Jiang, Yu Li, Yuan Rao, Jiayan Shao, Ying Liu, Lei Tian, Yicheng Mao, Xia Wang

**Affiliations:** a College of Food and Biological Engineering, Xihua University; Food Microbiology Key Laboratory of Sichuan Province, Chengdu 610039, China; b Sichuan Tujiu Liquor Co., Ltd, Nanchong 637919, China; c Chongqing Jiangji Distillery Co., Ltd, Jiangjin, 402260, Chongqing, China; d Xinjiang kaiduhe Liquor Co., Ltd, Xinjiang Uygur Autonomous Region, 830000, China

**Keywords:** Composite mold-enhanced *Xiaoqu*, Mixed-culture fermentation, *Qingxiangxing baijiu*, Alcohol yield, Flavor compounds

## Abstract

*Xiaoqu Qingxiangxing Baijiu* is typically produced using *Rhizopus* to make *Jiuqu*, but its alcohol yield and flavor are often unsatisfactory. This study innovatively employed a mixed-culture fermentation method to enhance its production. By screening three mold strains with high enzymatic activity (saccharifying enzymes, cellulase, and liquefying enzymes), a composite mold-enhanced *Xiaoqu* F1 (*Rhizopus oryzae* U1: *Aspergillus piperis* W3 = 2:1) was developed. F1 improved key physicochemical indicators (saccharifying, esterifying, and liquefying power) and microbial diversity in *Zaopei*. GC–MS analysis showed increased levels of flavor compounds (ethyl acetate, ethanol) and health-promoting factors (acetic acid, propionic acid, furans). Alcohol yield and flavor are often unsatisfactory. Yield rose by 2 %, and sensory tests noted reduced acridity and bitterness, with enhanced floral and fruity notes. This breakthrough in starter-making techniques offers new insights and theoretical support for improving *Baijiu* quality using composite mold-enhanced *Xiaoqu*.

## Highlights

• Drawing from scarce literature, this paper delves into mold's enhanced use on *Xiaoqu*.
• This study employed mixed-culture fermentation to produce *Xiaoqu*.
• This study assessed physicochemical properties of 2 commercial *Xiaoqu*.
• The study compared *Xiaoqu*'s physicochemical properties with or without added yeast.
• This study analyzed the correlation of physicochemical factors and microorganisms.

## 1 Introduction

Chinese *Baijiu* has a long history and is categorized into 12 aroma types. Among them, *Qingxiangxing Baijiu* is widely popular for its purity, sweetness, harmony, refreshing taste, and lingering aftertaste. However, compared to other types of *Baijiu*, it exhibits relatively low content and limited variety of flavor compounds, including esters, alcohols, acid compounds, etc. Therefore, this study aims to enhance the flavor profile of *Qingxiangxing Baijiu* by increasing the content of its primary flavor compound, ethyl acetate, while also elevating the levels of alcohol compounds to improve the overall quality of *Qingxiangxing Baijiu*. Both the alcohol yield and flavor are critical indicators for assessing the quality of *Baijiu*.

Alcohol yield is a crucial economic and technical indicator in *Baijiu* brewing. Enhancing the alcohol yield not only improves the efficiency of raw material conversion but also reduces production costs. Currently, experts have achieved remarkable results in improving the alcoholic yield of raw materials by selecting high-quality yeast (Weimin et al., 2013). However, the application of molds in *Baijiu* brewing remains limited. As the most crucial microorganism in the fermentation process of *Baijiu* (Wang et al., 2022), molds produce a diverse array of enzymes such as proteases, lipases, glucoamylases, and cellulases. These enzymes possess capabilities such as aldolization and esterification, and the growth of mold mycelium facilitates water evaporation and material utilization within the liquor starter, playing a vital role in its maturation. Importantly, the quantity and activity of saccharifying enzyme produced by molds have been shown to positively correlate with the improvement in alcoholic yield of raw materials (Fu et al., 2021).

Presently, factory-produced *Jiuqu* primarily relies on the cultivation of a single type of mold, which often results in suboptimal saccharification efficiency. Mixed-culture fermentation can take advantage of the synergistic effect of enzyme systems between microorganisms to effectively improve enzyme activity and thus increase the yield of *Baijiu*. Therefore, this study mainly adopts a composite mold approach for the production of *Xiaoqu*. During mixed cultivation, molds demonstrate notable “cohesiveness”, allowing them to coexist with different strains of molds on the fermentation agent matrix through mechanisms such as symbiosis and antagonism. Not only do they coexist harmoniously, but they also have the ability to stand out, with the most prominent example being the “coexistence” of *Rhizopus* and *Aspergillus* (Zhang Donglin, 1999). This study will contribute to enhancing the saccharifying power of *Xiaoqu*, improving the alcohol yield of *Qingxiangxing Baijiu*, enhancing flavor, and providing a new approach for the production of high saccharification *Xiaoqu*.

## 2 Materials and methods

### 2.1 Sample collection

Fungi strains: *Rhizopus oryzae* U1, *Lichtheimia ramosa* W1, *Rhizopus arrhizus* W2, *Aspergillus piperis* W3, *Aspergillus unguis* W4, and *Aspergillus flavus* W5 were obtained from the Food Microbiology Key Laboratory Preservation Center at Xihua University in Sichuan Province; Industrial yeast was sourced from Sichuan Tujiu of *Baijiu* Industry Co., Ltd. Huaxi *Xiaoqu*, Anqi *Xiaoqu*, and bran were all purchased from the Hongguang Agricultural Market in Pixian District, Chengdu City, Sichuan Province. CK(J) *Xiaoqu* and CK *Xiaoqu* were provided by the brewing workshop of Sichuan Tujiu of *Baijiu* Industry Co., Ltd.

### 2.2 Enzyme activity measurement

The method for determining saccharifying enzyme activity, acidic protease activity, cellulase activity, lipase activity and liquefying enzyme activity of mold is determined according to *the Baijiu Production Technology Complete book* (Shen Yifang, 1998).

### 2.3 Physicochemical analysis of *Xiaoqu*

The moisture content, acidity, liquefaction ability, starch content, esterification ability, saccharification ability and fermentation ability of *Xiaoqu* were determined using “General methods of analysis for *Daqu*” (QB/T4257–2011, 2011). The pH of was measured using a pH meter, and the number of molds was determined using a hemocytometer counting method (Ning Zhengxiang, 2001).

### 2.4 *Xiaoqu* production

#### 2.4.1 Triangle flasks to expand the culture

We used triangular flask containing bran medium. The loading amount was 50 g of dried bran per 250 mL triangular flask. The molds preserved on the slant were separately inoculated into the triangular flask with bran medium. Subsequently, 80 % distilled water was added, and the mixture was incubated at a constant temperature of 30 °C for 32–48 h.

#### 2.4.2 Preparation of shallow pan bran *Xiaoqu*

A single pan was loaded with 200 g of bran (calculated on a dry basis), mixed with 80 % distilled water. The triangular flask mold *Jiuqu* (2.4.1) is inoculated into the pan. The total amount of mold inoculum was 0.6 % (calculated on a dry basis of bran). The mixture was cultured at 28–30 °C for 2–3 days, and every 4–6 h after the initial 8-h period, the surface of *Xiaoqu* was turned once. After cultivation, *Xiaoqu* was dried at 40–50 °C. Then, it underwent sieving using a 30-mesh. Subsequently, industrial yeast was mixed in at a ratio of 10 % (calculated on a dry basis of bran), stirred thoroughly, and stored in a cool and ventilated place. The steps for producing *Xiaoqu* are illustrated in Fig. S1.

At the same time, a control group CK(J) was established, consisting of the finished *Xiaoqu* produced using only a single strain of *Rhizopus* (Q303) and industrial yeast.

### 2.5 Determination of flavor compounds

#### 2.5.1 Determination of flavor compounds in *Zaopei*

The samples of *Zaopei* of the experimental group and the control group on the first, fourth and seventh days of *Baijiu* fermentation were collected respectively. Three samples were taken in parallel every day.

Accurately weigh 2 g of fermentation mash into a 15 mL headspace vial, then add 5 mL of distilled water and 3 g of sodium chloride. Mix well, then add 20 μL of 2-octanol at a concentration of 0.822 mg/mL as an internal standard (Song et al., 2019). Flavor compounds in *Zaopei* were extracted and identified using headspace solid-phase microextraction coupled with gas chromatography–mass spectrometry (HS-SPME-GC–MS) (Xu et al., 2023).

A semi-quantitative analysis of flavor substances was performed according to formula 2.1.

(2.1) $$C_{1}=C_{2}\times \left(A_{1}/A_{2}\right)$$

In the formula: $C_{1}$ represents the concentration of flavor compounds in *Zaopei*, $C_{2}$ represents the final concentration after adding to the headspace vial, $A_{1}$ represents the peak area of flavor compounds in *Zaopei*, and $A_{2}$ represents the peak area of the internal standard 2-octanol.

#### 2.5.2 Determination of flavor compounds in the original *baijiu*

Samples of the original *Baijiu* were collected on the 7th day of fermentation from both the experimental group and the control group. Three parallel samples were taken, resulting in a total of six samples. They were labeled as S-1, S-2, S-3 for the experimental group and D-1, D-2, D-3 for the control group.

Precisely measure 5 mL of the original *Baijiu* sample into a 15 mL headspace vial. Add 3 g of NaCl and 5 uL of the internal standard (2-octanol with a concentration of 822 mg/mL) to the vial. The specific steps for determining flavor compounds in the original *Baijiu* and the semi-quantitative calculation formula can be referenced from Section 2.5.1.

### 2.6 Determination of alcohol yield

The alcohol yield refers to the volume or weight of *Baijiu* with an alcohol degree of 50 in a unit of raw material produced under standard atmospheric pressure and at 20 °C. The formula for calculating alcohol yield is as follows: Alcohol yield = (original alcohol degree/ 50) × original *Baijiu* weight/ raw material weight.

### 2.7 Sensory analysis

Quantitative descriptive analysis was carried out by 10 panelists (5 men and 5 women) with sensory analysis experience. Tests were conducted in individual cubicles and samples were presented in a randomized order. The aroma descriptors perceived included eight properties: caramel flavor, acrid taste, bitter taste, sour taste, smoky aroma, floral aroma, sweet taste and fruity aroma. Panelists were required to evaluate the samples on the basis of a 5-point intensity scale from 0 (not perceivable) to 4 (strongly perceivable). The personnel in the panel were trained before the test to describe the intensity scaling of aroma, flavor, and mouthfeel attributes (S. Y. Sun et al., 2018).

### 2.8 Statistical methods

Line graphs and scatter plots were generated using GraphPad Prism (version 9.0.3); bar graphs were obtained using Origin 2018 software. Circos plots at the species level and bar graphs were analyzed and created using Circos v0.66–7 and Python 2 (matplotlib-v1.5.1), respectively. VIP heatmaps and correlation network plots were created using Meta Analyst and the Lianchuan Biological Cloud Platform. Spearman correlation and Mantel tests were visualized using the “linkET” package in R (v4.1.1) software.

## 3 Results and discussion

### 3.1 Enzyme production capability assay

**Fig. 1:**
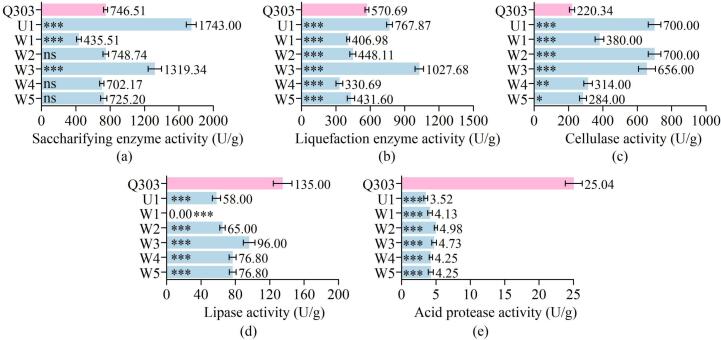
Enzymatic activities of mold. Q303 represents pure *Rhizopus* inoculated in traditional *Xiaoqu* in the factory. Note: (a) U/g represents 1 unit of enzyme activity, defined as the amount of enzyme that produces 1 mg of glucose per hour from 1 g of dried mold under conditions of 40 °C and pH 4.6. (b) U/g represents 1 unit of liquefying enzyme activity, defined as the amount of enzyme that liquefies 1 g of soluble starch per hour under conditions of 35 °C and pH 4.6 from 1 g of dry mold. (c) U/g represents 1 unit of cellulase activity, defined as the amount of enzyme that releases 1 μg of glucose in 30 min from 1 g of bran in each milliliter of the fermentation supernatant using carboxymethyl cellulose sodium liquid medium as the culture medium. (d) U/g represents 1 unit of lipase activity, defined as the amount of enzyme that hydrolyzes 1 μmol of fat per minute from 1.0 g of solid *Xiaoqu* powder under conditions of 40 °C and pH 7.5. (e) U/mL represents 1 unit of acidic protease activity, defined as the amount of enzyme that hydrolyzes casein to produce 1 μg of amino acid per minute from 1.0 g of solid dry mold under conditions of 40 °C and pH 3. *Represents *P*<0.05; **represents *P*<0.01; ***represents *P*<0.001 (*n* = 3).

Using saccharifying enzyme activity, liquefying enzyme activity, cellulase activity, acidic protease activity, and lipase activity as indicators, the enzyme activities of six mold strains were measured, with Q303 as the control. As shown in Fig. 1, the saccharifying enzyme activities of U1, W3, and W2 reached 1743 U/g, 1319.34 U/g, and 748.74 U/g, respectively, all exceeding that of Q303 (746.51 U/g). Moreover, these three strains also demonstrated significant advantages in cellulase activity measurements (Fig. 1c).

*Rhizopus* is capable of secreting large amounts of amylase and glucoamylase, hydrolyzing starch polysaccharides to enhance saccharification (Suprianto et al., 1989). *Rhizopus* can grow in low-moisture media while undergoing both growth and saccharification processes (Liu & Lien, 2016). This makes it suitable for the production of alcoholic beverages (Johnson et al., 2009) or syrups through solid-state or semi-solid-state methods. In *Baijiu* production facilities, the conclusion of solid-state saccharification depends on the conversion rate of starch to glucose. These advantagesf = make *Rhizopus* a commonly used “starter” for solid-state fermentation in the production of alcoholic beverages.

Liquefying enzymes can hydrolyze starch-based raw materials into nutrients such as dextrins, maltotriose, linear maltose oligosaccharides, and glucose, which are essential for microbial growth (Gupta et al., 2003). Therefore, higher liquefying enzyme activity plays a significant role in promoting the *Baijiu* brewing process. As shown in Figure (Fig. 1b), only W3 (1027.68 U/g) and U1 (676.87 U/g) exhibited higher liquefying enzyme activity than Q303 (570.69 U/g), while the enzyme activities of the other strains were all below 500 U/g.

In the determination of lipase activity (Fig. 1d), Q303 exhibited the highest activity (135 U/g), and among the other strains, W3 showed relatively high activity (96 U/g), while W1 had almost no activity. Q303 also demonstrated the highest acid protease activity (25.04 U/g), whereas the acid protease activities of the other six strains were lower and relatively similar, ranging between 3–5 U/g (Fig. 1e).

Following a comprehensive evaluation, the strains W2, W3, and U1 were selected as the inoculation strains for the subsequent *Xiaoqu* production due to their high saccharification, liquefaction, and cellulase activities.

### 3.2 The physical and chemical characteristics of *Xiaoqu*

The biochemical characteristics of *Xiaoqu* determine the quality and fermentation suitability of the starter (Fan et al., 2018).

**Fig. 2:**
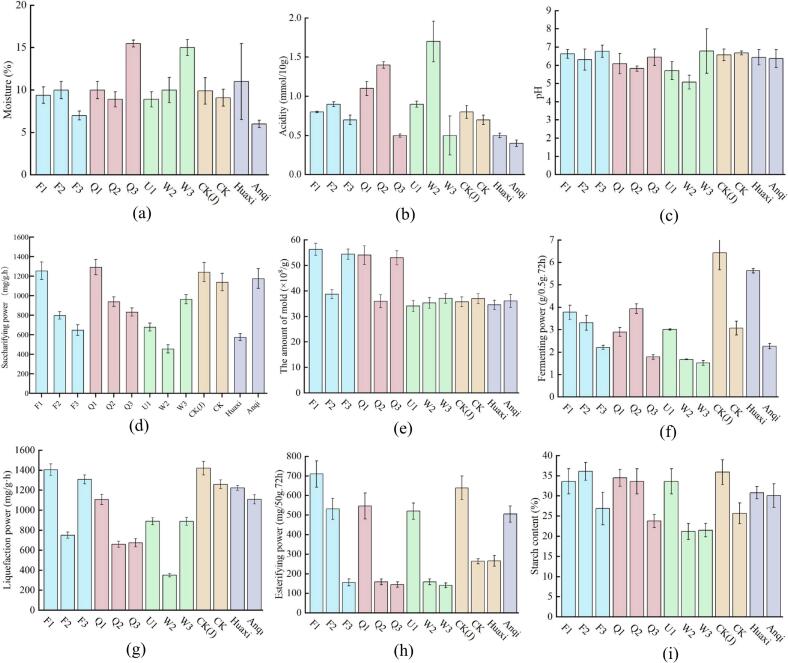
The differences in the physicochemical properties of *Xiaoqu* inoculated with different combinations of molds. (a) Moisture content, (b) Acidity, (c) pH, (d) Saccharifying power, (e) The amount of mold, (f) Fermentating power, (g) Liquefaction power, (h) Esterifying power, (i) Starch content. Note: F1, F2, F3 represent U1:W3 = 2:1; U1:W3 = 1:1; U1:W3 = 1:2 respectively; Q1, Q2, Q3 represent W2:W3 = 2:1; W2:W3 = 1:1; W2:W3 = 1:2 respectively. CK(J) represents the control *Xiaoqu* with yeast produced by the distillery's fermentation process. CK represents the control *Xiaoqu* without yeast from the distillery.

Moisture content: Moisture content is closely related to microbial growth, and microbial growth is essential for the safe storage of *Xiaoqu* (Kang, Jia, et al., 2022). Generally, the safe threshold for moisture content in mature *Xiaoqu* used for *Baijiu* fermentation is approximately 13 % (g/100 g) (Penghui Liu et al., 2019). As shown in Fig. 2a, except for Q3 and W3 with moisture contents of 15.5 % and 15.0 %, respectively, the moisture content of other *Xiaoqu* is below 13 %. The lower moisture content of *Xiaoqu* after the fermentation process indicates increased production and evaporation of free moisture during fermentation, leading to better maturity of the *Xiaoqu* (Penghui Liu et al., 2019).

Acidity and pH: The acidity of *Jiuqu* is mainly related to the metabolism of lactic acid bacteria (LAB) and other acid-producing microorganisms, as well as the degradation of fats, starch, and proteins (Kang, Sun, et al., 2022). Generally, the acidity of good *Jiuqu* should not exceed 1.0 mmol/10 g. According to Table S1, the acidity of Q1, Q2, and W2 all exceeded 1.0 mmol/10 g. Therefore, these three *Xiaoqu* samples were excluded.

Saccharifying power: As shown in Fig. 2d, Q1 and F1 exhibit the highest saccharification efficiency, with values of 1293 mg/g·h and 1256 mg/g·h, respectively, which are 51 mg/g·h and 14 mg/g·h higher than CK(J). Furthermore, the saccharification efficiency of F1, which undergoes mixed-culture fermentation, is approximately twice as high as that of single-culture U1 (679 mg/g·h) and W2 (456 mg/g·h). Similarly, Q2 exhibits a saccharification efficiency approximately 450 mg/g·h higher than that of single-culture W2.

The amount of mold: Referring to Fig. 2e, compared to *Xiaoqu* cultivated with single molds, those produced through composite mold cultivation, such as F1 (56.31 × 10^8^ cells/g), F3 (54.40 × 10^8^ cells/g), Q1 (54.07 × 10^8^ cells/g), and Q3 (52.97 × 10^8^ cells/g), have higher mold counts. This indicates that *Rhizopus* and *Aspergillus* strains do not exhibit purely competitive inhibition; instead, they can engage in mutually beneficial symbiosis to some extent (Li Hongyuan et al., 2021). The saccharification efficiency of F1 and Q1 *Xiaoqu* exhibits a positive correlation with the mold count, indicating that an increase in mold count can enhance the saccharification efficiency of *Xiaoqu* to some extent.

Fermentating power: In comparison to CK(J), CK (1140 mg/g·h) does not show significant differences in saccharification efficiency, suggesting that yeast does not contribute significantly to improving saccharification efficiency. However, the role of yeast is highly significant in terms of fermentation efficiency (Fig. 2f). CK(J) with added yeast exhibits a fermentation efficiency of 6.43 g/0.5 g·72 h, which is approximately twice as high as that of CK without added yeast (3.07 g/0.5 g·72 h). According to previous research, fungi primarily utilize starch and other raw materials to convert them into sugars using amylase, while a significant portion of yeast indirectly utilizes these sugars to produce carbon dioxide or ethanol (Su et al., 2020). However, some studies have also mentioned that certain starch-degrading yeast can secrete large amounts of α-amylase, glucoamylase, acid protease, and β-glucosidase, which can synergistically degrade starch along with fungi, thereby improving the utilization of raw materials (Wang et al., 2021).

Liquefaction power: As shown in Fig. 2g, the liquefaction levels of F1 (1405 mg/g·h), F3 (1309 mg/g·h), Q1 (1107 mg/g·h), CK(J) (1421 mg/g·h), CK (1259 mg/g·h), Anqi (1109 mg/g·h), and Huaxi (1223 mg/g·h) all remain above 1000 mg/g·h, with F1's liquefaction level being comparable to that of CK(J).

Esterifying power: As shown in Fig. 2h, the esterifying power of the CK without yeast is very low, whereas esterifying power is typically contributed by yeast capable of producing aroma (Wang et al., 2011). In this study, the esterifying power of F1 (710 mg/50 g·72 h), F2 (532 mg/50 g·72 h), Q1 (546 mg/50 g·72 h), U1 (520 mg/50 g·72 h), Anqi (505 mg/50 g·72 h), and CK(J) (639 mg/50 g·72 h) are relatively high, reaching 500 mg/50 g·72 h, and F1's esterifying power is higher than that of CK(J).

Taking into account the comprehensive comparison of the above situations, F1 (U1:W3 = 2:1) is ultimately selected for further experiments because its indicators such as saccharifying power, esterifying power, reducing sugar content, etc., can maintain at medium to optimal levels, and its moisture content and acidity are within normal ranges.

### 3.3 The physicochemical properties of *Zaopei*

**Fig. 3:**
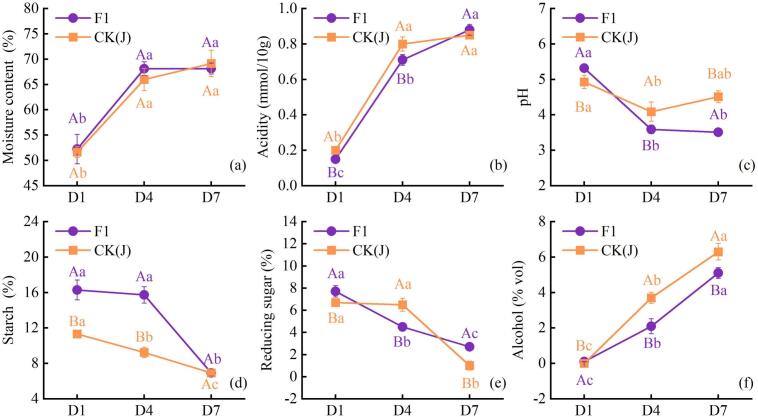
Physicochemical kinetics during fermentation of F1 and CK(J). Moisture content (a); Acidity (b); pH (c); Starch content (d); Reducing sugar (e); Alcohol (f). Letters A-C; a-c indicate significant differences, *P* < 0.05 (n = 3).

In *Baijiu* brewing, moisture content and acidity are typically considered critical environmental variables. They not only reflect the fermentation process together with indicators like reducing sugars and starch (Li et al., 2016) but also closely correlate with microbial activity, playing a crucial role in ensuring the quality of the base liquor (Wang et al., 2011). As shown in Fig. 3, the moisture content rapidly increased from days 1 to 4, with both F1 and CK(J) rising from around 50 % to about 70 %. Day 4 flattens out. During the fermentation process, an increasing moisture content indicates a more normal and thorough fermentation of *Zaopei*. The acidity rapidly increases in the early fermentation stage, from 0.1 % to 0.2 % to around 0.7 % to 0.8 % for both F1 and CK(J) within the first 1–4 days. In the later stages, the increase levels off but still shows a slight upward trend. A certain level of acidity promotes the proliferation of acid-resistant microorganisms such as LAB (Ma et al., 2022), accelerating the gelatinization and saccharification of grains, suppressing the growth of harmful microorganisms, and contributing to the taste of the *Baijiu* (Li et al., 2016).

Regarding the changes in starch, as shown in Fig. 3d, the starch content of F1 reached its highest level on the 1st day of fermentation, at 16 %, which was more than 4 % higher than that of CK(J). As fermentation progressed, the starch content of CK(J) decreased almost linearly, while that of F1 decreased more gradually during the first 1–4 days and then decreased rapidly during the 4–7 days, ultimately both reaching around 6 %. Microorganisms utilize amylases to convert starch into glucose (Han et al., 2023). In the early stages of fermentation, when the temperature is low and not conducive to bacterial growth, the population of starch-degrading bacteria such as *Bacillus* is relatively low (Xu et al., 2023), which is one of the reasons for the relatively minor changes in starch content during the early stages of fermentation.

The trend of reducing sugar varies slightly among different samples (Fig. 3e). In CK(J), there is a slight increase in reducing sugar during the first 1–4 days of fermentation. It is speculated that during the early stages of fermentation, fungi may play a dominant role, leading to rapid saccharification and a rapid decrease in starch concentration, resulting in a rapid increase in reducing sugar content to around 7 %. As fermentation progresses and the population of yeast and other microorganisms increases (Xu et al., 2023), fermentation activity strengthens, leading to a sharp decrease in reducing sugar content. The differences in microbial content among samples, with variations in growth rates, result in different dynamic changes in their physicochemical properties. The alcohol content on the first day of fermentation is around 0 for both samples, but increases rapidly as fermentation progresses. CK(J) reaches around 6 % alcohol content by the 7th day, while F1, although slightly lower, also reaches around 5 %. When the concentration of alcohol or acidity is relatively high (days 4–7), the growth of yeast is inhibited (Liu et al., 2017). Additionally, during this stage, a significant amount of heat is generated due to microbial degradation of organic matter (Wei et al., 2018), resulting in an increase in fermentation temperature. This elevated temperature promotes the proliferation of alcohol-tolerant bacteria, accelerating alcohol production. Starch serves as the substrate for alcohol fermentation, and during the process of alcohol production, the starch content also decreases rapidly (d), especially during days 4–7, with reductions of 11 % and 4 %, respectively. In the fermentation process of *Baijiu*, there exists a complex interplay and mutual constraint among temperature, moisture content, acidity, starch, reducing sugars, and alcohol. Generally, the greater the difference in moisture content between the start and end of fermentation, the faster the decline in starch concentration in the mash, resulting in lower levels of reducing sugars and higher alcohol content. This indicates a more thorough fermentation process and higher alcohol yield. However, the deeper connections among these factors require further exploration. In this study, both F1 and CK(J) exhibited a rapid decrease in starch concentration, with a significant difference in moisture content before and after fermentation. The rapid increase in alcohol content suggests high microbial utilization efficiency, thorough fermentation, and ultimately contributes positively to the improvement of *Zaopei*.

### 3.4 Diversity and composition of microorganisms

**Fig. 4:**
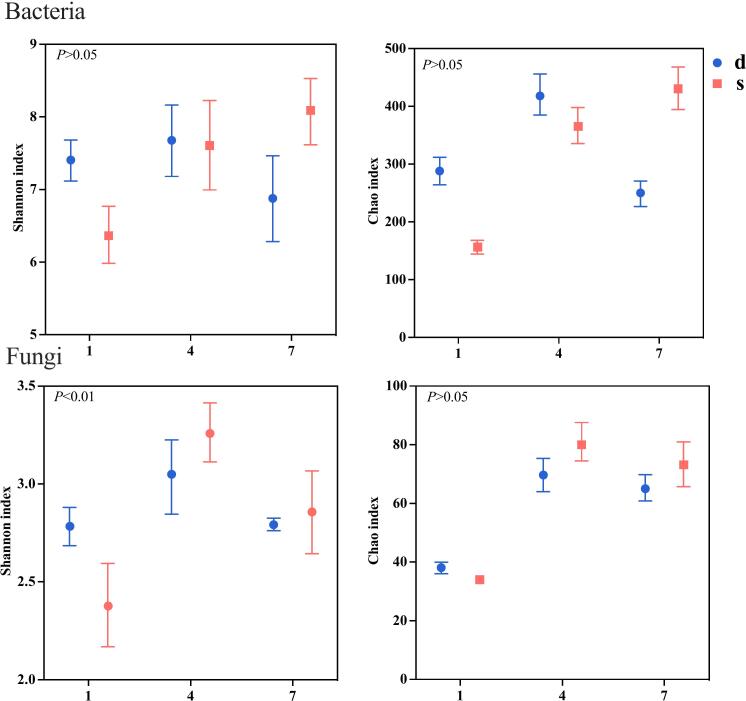
Microbial diversity of *Zaopei* from F1 and CK(J) on days 1, 4, and 7 of fermentation. Independent sample *t*-test results (*P*) are shown in the figure.

The treated *Zaopei* samples were subjected to qPCR and amplicon sequencing analysis to explore the dynamic characteristics of the microbial community. The sequences obtained from 18 *Zaopei* samples were classified into operational taxonomic units (OTUs) at 97 % similarity and used to calculate Shannon and Chao indices (Fig. 4). As fermentation days increase, the Shannon index of bacteria in F1 gradually increases and surpasses that of CK(J) by day 7 of fermentation. A similar trend is observed in the Chao index. Interestingly, F1 exhibited a distinct variation trend compared to CK(J), with statistically significant differences emerging by day 7 of fermentation. This could be attributed to the accumulation of acid-producing bacteria, such as *Acetobacter*, *Levilactobacillus*, and *Companilactobacillus*, creating an acidic environment during the mid-phase of fermentation in CK(J). This acidic environment may not be conducive to microbial growth, especially for aerobic microorganisms (Zhang et al., 2019), leading to a reduction in microbial diversity in the later stages of fermentation when *Zaopei* is undergoing complete anaerobic fermentation.

**Fig. 5:**
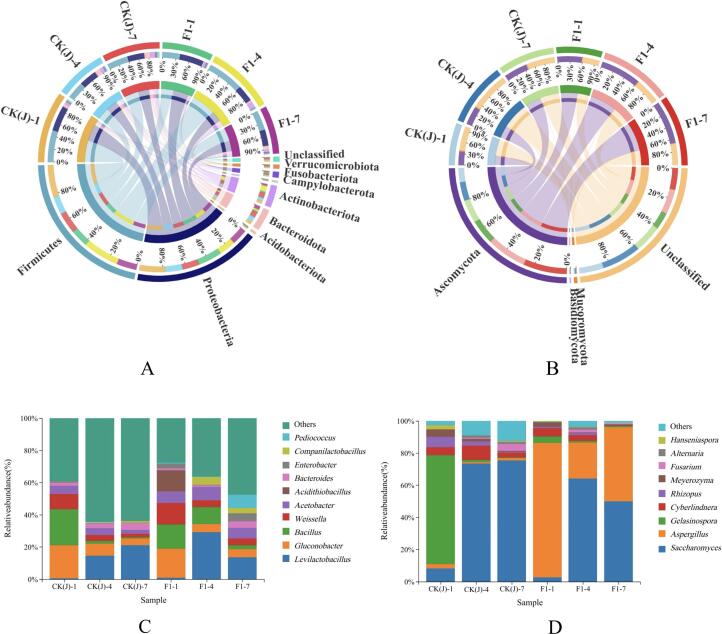
Distribution of bacteria (A) and fungi (B) at the phylum level, as well as changes in genera of primary bacteria (C) and molds (D) in *Zaopei* from F1 and CK(J) on days 1, 4, and 7 of fermentation (*P* < 0.05).

At the genus level (Fig. 5), the predominant bacterial genera are *Levilactobacillus*, *Gluconobacter*, *Bacillus*, *Weissella* and *Acetobacter*, while the dominant fungal genera include *Saccharomyces*, *Aspergillus*, *Gelasinospora*, *Cyberlindnera* and *Rhizopus.*

Notably, *Aspergillus* and *Saccharomyces* are the two most abundant genera in F1, serving as the key functional microbes for saccharification and ethanol production, respectively. Another noteworthy observation is the significant divergence in fungal diversity. Both groups peak on day 4 of fermentation, but by day 7, F1 exhibits slightly higher fungal diversity compared to CK(J). And *Aspergillus* maintains relatively high abundance until the final stage.

Additionally, *Bacillus* genus has the potential to produce cellulase and hemicellulase enzymes, contributing to the formation of volatile compounds such as diacetyl during *Baijiu* fermentation (Zhang et al., 2013). In previous research, Yang et al. combined multi-omics approaches such as metaproteomics to identify *Bacillus subtilis* as a microorganism that promotes the formation of a stable microbial ecosystem under specific environmental conditions. It regulates the metabolism of pentameric heterocyclic amino acids in *Daqu*, thereby influencing the differentiation of microbial ecology in *Jiuqu* (Yang et al., 2023). Several scholars have isolated strains of *Bacillus subtilis*, *B. velezensis*, *B. licheniformis*, and *B. amyloliquefaciens* from *Jiuqu* (Tang et al., 2023). They have incorporated *Bacillus* species as functional microorganisms into *Jiuqu* to produce fortified *Jiuqu*, aiming to improve the quality of *Jiuqu* and subsequently enhance the flavor and quality of *Baijiu*. This approach provides a new avenue for future research on mixed microbial fermentation, ultimately aiming to increase the yield of *Jiuqu*. He et al. (2019) added *Bacillus velezensis* and *Bacillus subtilis* to produce fortified *Jiuqu*, resulting in a relative increase of 6.2 %, 5.3 %, and 16.8 % in the abundance of *Bacillus*, *Lactobacillus*, and *Candida* species, respectively. The liquefying, saccharifying, and esterifying power of fortified *Daqu* increased by 25.6 %, 9.0 %, and 15.2 %, respectively. Moreover, the content of ligustrazine increased by 1.9 times, and the abundance of enzyme-coding genes involved in saccharification, alcohol fermentation, and aroma generation significantly increased (He et al., 2019).

At the phylum level, circos analysis indicates that *Firmicutes*, *Proteobacteria*, *Actinobacterota*, and *Bacteroidota* represent the main bacterial phyla. *Ascomycota*, *Mucoromycota*, and *Basidiomycota* are the main fungal phyla.

Notably, the saccharifying fungi *Ascomycota* consistently surpasses CK(J) in F1, and the increase in its quantity significantly enhances the alcohol yield (Fig. 5B).

Additionally, many studies have found the presence of *Actinobacteria* and *Bacillus* in *Huangjiu* (Liu et al., 2015), soy sauce (Hao & Sun, 2020), and strong aroma *Daqu* (He et al., 2019). They are also dominant bacteria in *Jiuqu* (Zhao et al., 2020). *Thermophilic actinomycetes* are a genus within *Actinobacteriota*, capable of thriving in harsh environments and producing various thermostable enzymes such as amylase, serine protease, protease, and lipase, with metabolic activity at 45–50 °C (Li et al., 2017).

### 3.5 Differences in flavor compounds of F1 and CK(J) *Zaopei*

The flavor compounds in *Baijiu* are endogenous substances produced during the production, saccharification, fermentation, distillation, and storage processes of *Baijiu*. The characteristic flavor compounds of *Baijiu* are an important basis for identifying *Baijiu* quality (Fan & Qian, 2006). As shown in Table S2, the flavor compounds of *Zaopei* were identified for both F1 and CK(J) during the fermentation of *Baijiu*, with a total of 185 compounds identified. These include 39 alcohols, 59 esters, 27 acids, 13 ketones, 10 aldehydes, 9 phenols, 21 hydrocarbons, 4 furans, 1 sulfide, and 2 other compounds. *Qingxiangxing Baijiu* is characterized by fruity, floral, sweet, and cereal aromas, with ethyl acetate being the primary flavor compound (Wang et al., 2020). Its concentration reached up to 4.51 mg/kg in F1 and 4.31 mg/kg in CK(J). Although ethyl acetate was not detected in F1 on the first day of fermentation, its content gradually increased over time, and by the 7th day of fermentation, F1 showed an increase of 0.2 mg/kg compared to CK(J). The presence of ethyl acetate is crucial for maintaining the clean aroma style of *Baijiu*. Conversely, ethyl lactate, known for their fruity and slight fatty notes, contribute to the length of aftertaste. Typically, an appropriate ratio between the two (1: 0.6) results in an elegant, smooth, or aromatic flavor profile. In F1–7, the ratio of ethyl acetate to ethyl lactate is approximately 1: 0.5, enhancing the aroma complexity of *Baijiu*. lsopentyl acetate was detected in both CK(J)-7 and F1–7, with its highest concentration reaching up to 4.57 mg/kg in CK(J), imparting a unique banana aroma (Quilter et al., 2003). Gamma-Nonanolactone, also known as coconut aldehyde, is a compound with a coconut aroma (Wang, Chang, et al., 2021). Detected on CK(J)-4 and CK(J)-7, this substance is primarily composed of a five-membered lactone heterocycle. Due to its pleasant odor, it finds wide applications in the food and beverage industry (Sun et al., 2019). We were pleasantly surprised to detect the presence of the uniquely sweet-rose-scented compound, acetophenone, in the CK(J) fermentation on the 7th day. Besides its aroma and taste, it also possesses valuable biological properties, demonstrating anti-trypanosomal activity and showing strong repellent effects against ticks. It acts as a deterrent against the bark beetle in Asian larch trees (Bero et al., 2013) and serves as a repellent for the Mediterranean fruit fly (Bonikowski et al., 2015). Additionally, it plays a crucial role in influencing cherry aroma (Sun et al., 2010) and may be a determining factor in the quality of green tea. Furthermore, Furfuryl alcohol, which carries a sweet and honey-like taste, was detected in both F1 and CK(J) groups. The sulfur compound diallyl disulfide, mainly found in garlic, has been detected at levels around 0.01–0.02 mg/kg (Kim et al., 2012). It has been found to have potential applications in various fields, such as being used as a fumigant to inhibit the reproduction of grain pests (Yang et al., 2012), inducing apoptosis in cancer cells, and exerting anticancer effects (Saidu et al., 2012). In a study by Iciek, Malgorzata B. et al., diallyl disulphide was also found to have antioxidant effects in the liver, forming sulfur-containing compounds, which could be used to protect normal liver cells during chemotherapy or alleviate liver damage (Iciek et al., 2012).

**Fig. 6:**
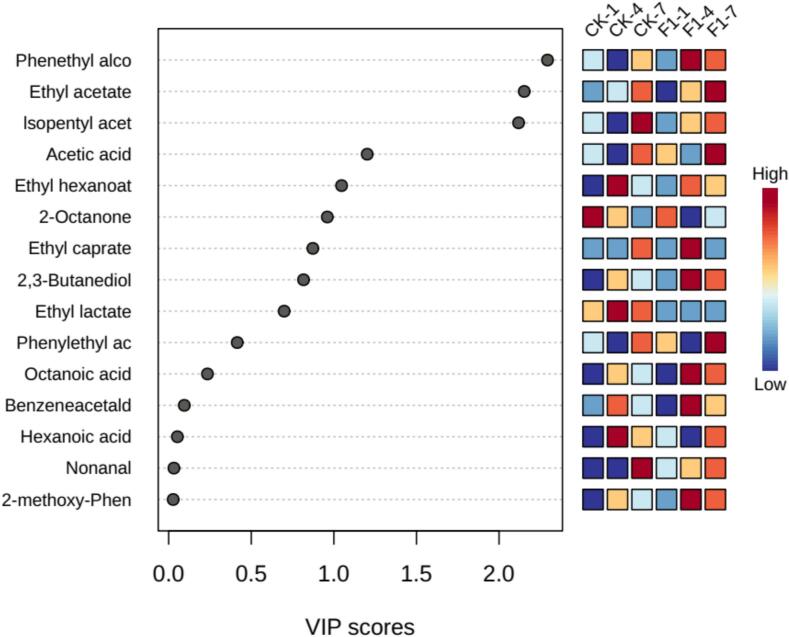
F1 and CK(J) *Zaopei* heatmap visualization of volatile compounds on days 1, 4, and 7. The colored boxes on the right indicate the relative concentrations of the corresponding flavor compounds in each group under study.

The importance of 20 different volatile compounds, including 3 alcohols, 3 aldehydes, 7 esters, 3 acids, 2 phenols, and 2 ketones, was determined using the Variable Importance in Projection (VIP) method. Detailed VIP values are provided in Table S3. Here, the VIP value of phenethyl alcohol is the highest, at 2.29, followed by ethyl acetate, which is 2.15. Some microorganisms, such as *Aspergillus* and yeast, can produce alcohol compounds like phenethyl alcohol through lipid oxidation (Tang et al., 2019). The top 15 most significant compounds are illustrated in Fig. 6. At day 4 of fermentation, CK(J) exhibits a higher abundance of lipid compounds, with *Saccharomyces* being the dominant genus on day 4 (Fig. 5D), which likely produces a significant amount of lipid compounds through biosynthesis. At the end of fermentation, the main volatile compounds in CK(J) are isoamyl acetate and nonanal, while F1 contains ethyl acetate, acetic acid, and Phenethyl alcohol. Phenethyl alcohol is found in various plants such as apple, almond, banana, rose, hyacinth, jasmine, and lily (Zhou et al., 2019), providing white wine with a creamy, rose-like aroma (Tang et al., 2023). In the amino acid branch, phenylethanol originates from the metabolism of phenylalanine through the Ehrlich pathway in yeast (Rahayu et al., 2017). Additionally, benzaldehyde and Phenethyl alcohol can serve as precursors for aromatic compounds.

### 3.6 Microbial-environmental factors and the correlation between microorganisms and flavor compounds

**Fig. 7:**
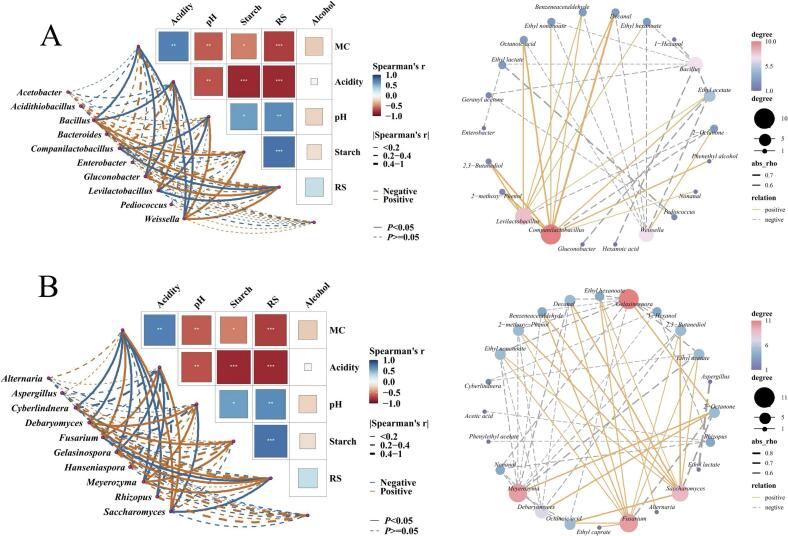
Integrated correlation analysis of microbiota-physicochemical factors-flavor compounds. A: Bacteria; B: Fungi. The width of the lines corresponds to the Spearman test-derived r-value, and the color of the lines represents positive or negative correlations. Solid and dashed lines represent positive and negative correlations, respectively, between microorganisms and physicochemical factors as determined by the Spearman test. The heatmap displays the Spearman correlations between each physicochemical factor.

As shown in Fig. 7, studying the correlation among environmental factors and the correlation between core microbial communities in *Xiaoqu*, and environmental factors, mathematical models can be established based on experimental data. This helps in achieving the goal of controlling microorganisms by controlling fermentation process parameters. Furthermore, by calculating Spearman correlation coefficients, selecting coefficients (|rho| > 0.5), and significant relationships (*P* < 0.05) between dominant genera (top 10 species abundance) and quantitative flavor compounds as relevant nodes, a network diagram is created.

*Rhizopus* shows a significant negative correlation with acidity, while *Saccharomyces* exhibits a significant positive correlation with acidity (Fig. 7B). This can explain the slight increase in reducing sugars observed in CK(J) during days 1–4 of fermentation mentioned earlier. During the initial 1–4 days of fermentation, when acidity is relatively low, *Rhizopus* dominates and breaks down the raw materials into reducing sugars, while the contribution of yeast is relatively insignificant. However, in the later stages of fermentation, acidity reaches its peak (Fig. 3), and the quantity of *Saccharomyces* increases. *Saccharomyces* becomes predominant in breaking down reducing sugars, leading to a rapid decrease in reducing sugars. Additionally, *Saccharomyces* exhibits a significant positive correlation with moisture content. Therefore, moisture content and acidity together promote the growth of *Xiaoqu*, thereby enhancing the utilization of reducing sugars. *Saccharomyces* is one of the key factors influencing the flavor profile and product quality of *Baijiu*, and it exhibits a significant positive correlation with most lipids (Fig. 7B). As a lipid-producing microorganism, it directly synthesizes lipids intracellularly, involving two intracellular enzymes: acetyl-coenzyme A synthetase and alcohol acetyltransferase, which are the main pathways for the formation of ethyl acetate in *Baijiu* (Lee, Seo, & Young, 2021). It is speculated that the presence of *Saccharomyces* in the *Zaopei* may lead to a mutual inhibition pattern with reducing sugars, as *Saccharomyces* metabolizes glucose substrates into ethanol and carbon dioxide, as shown in Fig. 7B.

Reducing sugars are significantly positively correlated with starch content, and both reducing sugars and starch content are significantly negatively correlated with acidity. Meanwhile, acidity shows a positive correlation with *Levilactobacillus* and *Companilactobacillus* (Fig. 7A), which are acid-producing bacteria. Moderate acidity can promote the formation of esters, making the aftertaste of *Baijiu* more mellow (Zhang et al., 2019). However, excessive acidity may have a counterproductive effect, affecting microbial growth. In addition, *Levilactobacillus* and *Companilactobacillus* are also important aroma-producing bacteria in *Baijiu* (Li et al., 2023), with their abundance representing a significant proportion of the microbiota. Most aldehydes and lipids show a significant positive correlation with them, such as the important aroma compound ethyl acetate in *Baijiu*. In recent years, the discovery of the smallest *Lactobacillus* species to date was made in the fermentation pits of Luzhou Laojiao (Zhao et al., 2022). There is reason to believe that further exploration is needed for the development of its new species and unknown functions in *Baijiu*. Conversely, *Weissella* shows a negative correlation with most lipids and acids, consistent with the correlation with acidity in Fig. 7 results. Interestingly, throughout the entire graph, *Weissella* shows a unique positive correlation with 2-Octanone, but there is limited literature exploring the relationship between *Weissella* and ketone compounds, indicating the need for further research. *Bacillus* exhibits a negative correlation with acidity (Fig. 7A), which is consistent with the findings of Kang et al. (Kang, Sun, et al., 2022). Meanwhile, there is a significant negative correlation with most esters (Fig. 7A), which contradicts many studies (He et al., 2019). Such inconsistencies in the results are reasonable and attributed to the complexity of microbial interactions and enzyme systems in *Zaopei* (Xu et al., 2023). Overall, the composition of bacterial colonies in *Zaopei* is mainly determined by moisture, acidity, starch content, and pH, while the distribution of fungal communities is mainly determined by moisture, acidity, starch content, reducing sugar, and pH (Fig. S2).

### 3.7 Comparison of flavor components in F1 and CK(J) fermented *baijiu*

**Fig. 8:**
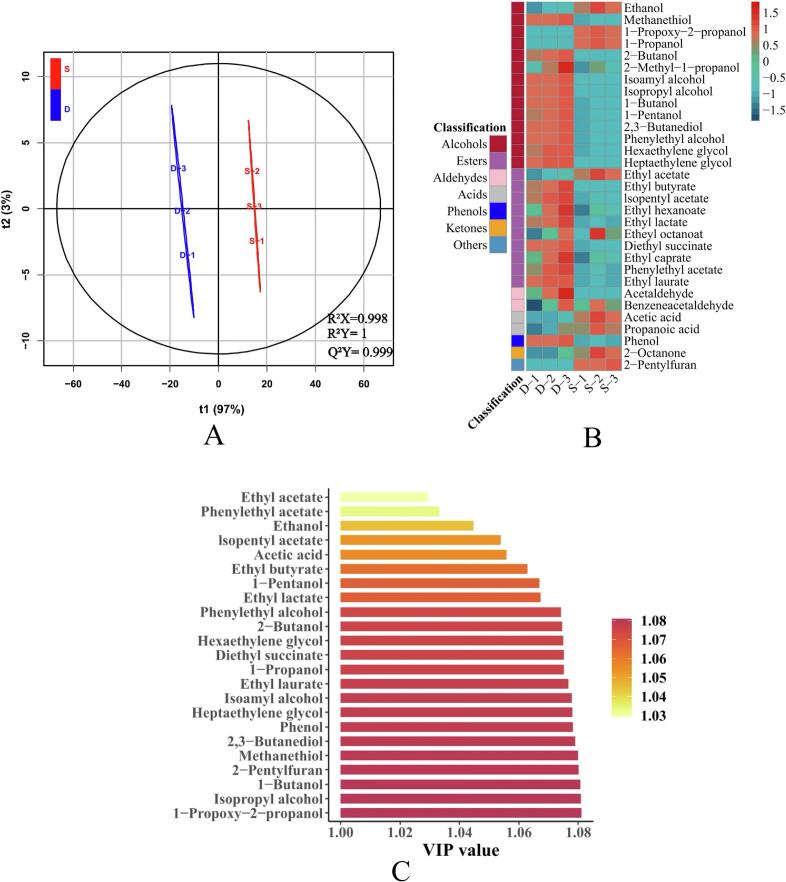
OPLS-DA score plot of volatile compounds in *Baijiu* fermented by F1 and CK(J) (A). Volatile profiles of *Baijiu* fermented by F1 and CK(J) (B); Data were standardized by the scale() function in R, with the formula Z = (X–u)/σ, where Z represents the standardized score, X is the variable, u is the mean, and σ is the standard deviation. Four significant features of the volatile profiles (C). S-1, S-4, and S-7 represent the *Zaopei* on the first, fourth, and seventh day of F1 fermentation, respectively; D-1, D-4, and D-7 represent the *Zaopei* on the first, fourth, and seventh day of CK(J) fermentation, respectively.

To comprehensively understand the differences in volatile components between F1 and CK(J), further analysis was conducted using Headspace Solid-Phase Microextraction-Gas Chromatography-Mass Spectrometry (HS-SPME-GC–MS) (Table S4). A total of 46 volatile compounds were detected and identified via GC–MS, and a heatmap was generated to describe their composition and abundance (Fig. 8B). Then, an Orthogonal Partial Least Squares Discriminant Analysis (OPLS-DA) model was established to differentiate the variable volatile compounds between F1 and CK(J) samples, as shown in Fig. 8A. The prediction score plot exhibited two well-separated groups (R^2^Ycum = 1), indicating the significant predictive efficacy of this model (Q^2^cum = 0.993) (Kang, Jia, et al., 2022). To assess the contribution of variables, the importance of variables in the projection (VIP) values was calculated through OPLS-DA analysis. Variables with VIP scores>1.0 and *P* < 0.05 were considered as significant contributors to the differences (Kang, Sun, et al., 2022). The important features of the 23 volatile compounds with VIP > 1 are shown in Fig. 8C, which were identified as significant volatile characteristics distinguishing between F1 and CK(J).

The OPLS-DA model identified the top 31 important volatile compounds used to distinguish between F1 and CK(J) (Fig. 8B). Among them, ethanol, 1-propoxy-2-propanol, 1-propanol, ethyl acetate, ethyl octanoate, acetic acid, propanoic acid, 2-octanone, and 2-pentylfuran contributed to the specificity of F1. F1 significantly increased the acetic acid content by 24.9 % (Fig. 8B). Acetic acid, as a health-promoting factor in *Baijiu*, is present in large amounts and is an important component that constitutes the typical characteristics of *Qingxiangxing Baijiu* (Chen et al., 2017). It has a strong sour aroma, slightly sour and sweet taste, and has effects such as prolonging lifespan, reducing inflammatory responses, and alleviating liver damage (Yang et al., 2019). Propionic acid imparts F1 original wine with a subtle, sweet, and clean aroma, sometimes with a hint of musty sweetness in taste and aroma. Generally, it is present in trace amounts in aromatic white wines (Guo Xuefeng et al., 2022). Research shows that *Baijiu* with higher levels of propionic acid and lactic acid play a crucial role in the comfort of mice after drinking (Guo Xuefeng et al., 2022). Enhanced fermentation also increases the production of furan compounds such as 2-pentylfuran. In a study by Jingke Liu et al., they utilized the Likens-Nickerson apparatus for simultaneous steam distillation/extraction of volatile compounds from rough rice (BM), milled millet (MM), and rice bran (MB), and found that 2-Pentyl furan dominated in rice bran (Liu et al., 2012). Interestingly, besides being an aroma compound, 2-Pentylfuran can also serve as a novel fruit fly repellent (Cha et al., 2021). Most importantly, F1 fermentation significantly increased the contents of ethanol and ethyl acetate by 8.9 % and 17.0 % respectively, both of which are essential flavor compounds in *Qingxiangxing Baijiu* (Pang et al., 2021). Strengthening microbial interactions in mold-enhanced *Xiaoqu* improves the saccharification effect of *Xiaoqu* and increases the content of esters. However, further exploration is needed on how to increase the variety of flavor compounds in high concentrations. The next target may involve the addition of other types of bacteria, such as *Bacillus* and *Actinomycetes*, to study the interactions between microbial communities and the quantity and types of enzymes involved.

### 3.8 Yield of *baijiu* fermented by F1 and CK (J)

The alcohol content of F1 and CK(J) base wines was measured as 61.6 %vol and 59.6 %vol respectively using the 2.6 alcohol measurement method. Calculating according to the formula for alcohol yield using 2.6, their alcohol yields were determined to be 58.52 % and 56.62 % respectively, indicating an increase of around 2 % in alcohol yield after the mold-enhanced *Xiaoqu* fermentation process.

Although there has been extensive research on the co-fermentation of yeast, such as the enhancement of enzyme activity and ethanol synthesis rate by *Saccharomyces cerevisiae* and Non-*S. cerevisiae*, improving the quality of *Baijiu* (Su et al., 2020), there is scarce literature exploring the effects of enhanced interaction among molds on *Baijiu*. This study lays the theoretical foundation for the increase in liquor yield and flavor by mold-enhanced *Xiaoqu*, providing feasibility evidence. The yield of liquor varies with different mixed formulations of microorganisms, and there are synergistic, antagonistic, or inhibitory effects among functional microorganisms during the brewing process of *Baijiu*. We cannot underestimate the impact of microbial growth and metabolism. Exploring the roles behind these microorganisms and further studying how to utilize multi-strain and multi-enzyme mixtures to enhance the yield of *Baijiu*, improve flavor, and enhance the quality require further research.

### 3.9 Sensory analysis

As shown in Fig. S3, the average intensity of some attributes of the distillate was perceived to be significantly different (*P* < 0.05), including acrid taste, bitter taste, sweet taste, fruity aroma, and smoky aroma. The results indicate that the application of F1 has reduced the pungent flavor and bitterness of *Baijiu*.

The content of aldehyde compounds, such as acetaldehyde, in F1 is lower than that in CK(J) (Table S4). Aldehyde compounds, represented by acetaldehyde, are the primary contributors to the pungent taste of *Baijiu*. Acetaldehyde has a pungent and drying aroma, and long-term consumption of *Baijiu* containing free acetaldehyde can lead to a dry throat after drinking. Additionally, the contents of isobutanol and isoamyl alcohol in F1 are relatively low (Table S3). These higher alcohols belong to the category of fusel oils. Specifically, 2-methyl-1-propanol has an extremely bitter taste, while isoamyl alcohol is slightly sweet with a hint of bitterness. Generally, *Baijiu* with high levels of fusel oils tends to have a pronounced bitter taste.

Conversely, this was accompanied by an increase in fruity aroma and sweet taste in F1. As previously mentioned, ethyl acetate contributes to the distinctive fruity and floral aroma of *Qingxiang Baijiu*. It is likely that the enhanced fruity aroma in F1 can be attributed to the increased content of ethyl acetate. The use of *Rhizopus oryzae* in the F1 is typical for the production of sweet *Jiuqu*, and a relatively high amount of *Rhizopus* was added in F1. *Rhizopus* primarily converts the starch, which is predominantly branched starch in grain, into soluble sugars. This may explain the more pronounced sweetness observed in the *Baijiu* produced with F1. In summary, the composite enhancement of *Xiaoqu* with mold has a positive effect on improving the sensory flavor profile of *Baijiu*.

## 4 Conclusion

By inoculating *Rhizopus oryzae* U1 and *Aspergillus piperis* W3 in a 2:1 ratio into bran, a composite fermentation was conducted to produce *Xiaoqu* with high saccharifying, liquefying, and esterifying power. This increased the microbial diversity of *Zaopei*, raised the levels of flavor compounds such as ethyl acetate and ethanol in *Qingxiangxing Baijiu*, and enhanced the production of health-promoting factors like acetic acid and furan compounds. Additionally, the alcohol production rate of grain has also increased by 2 % and it significantly reduced the acrid and bitter taste of traditional *Baijiu*, while enhancing floral and fruity aromas. This study represents a breakthrough in traditional *Xiaoqu* production technology by developing a composite mold starter culture. The innovation achieves synergistic optimization of ‘enzyme activity - flavor profile - alcohol yield,’ providing both novel methodologies and theoretical foundations for improving *Baijiu* quality through composite mold-enhanced *Xiaoqu*. For the next phase of experiments, to further investigate the synergistic effects between molds and yeasts, we plan to conduct strain combination optimization experiments and metabolic interaction mechanism analysis.

## Fundingss

This work was supported by the international innovation cooperation project of the Department of Science and Technology in Sichuan Province (Project No. 2025YFHZ0117), the Provincial Natural Science Foundation of Sichuan (Project No. 2024NSFSC2068), the Project of Xinjiang Kaiduhe Liquor Co., Ltd. (Project No. H232106), the Yibin science and technology Program (Project No. 2024SF018), and the Sichuan Tujiu Liquor Co., Ltd. (Project No. 222305).

## CRediT authorship contribution statement

**Tongwei Guan:** Project administration, Supervision, Writing – review & editing, Funding acquisition, Conceptualization. **Xinrui Yin:** Writing – original draft, Methodology, Data curation, Writing – review & editing. **Yuhang Jiang:** Investigation, Conceptualization, Validation. **Yu Li:** Validation, Funding acquisition. **Yuan Rao:** Resources, Conceptualization, Supervision. **Jiayan Shao:** Resources, Supervision. **Ying Liu:** Visualization, Validation. **Lei Tian:** Methodology, Supervision. **Yicheng Mao:** Software, Supervision. **Xia Wang:** Validation, Software.

## Ethical statement

The appropriate protocols were utilized to protect the rights and privacy of all participants during the execution of the research and were approved by the Ethics Committee of Xihua University. All participants provided verbal consent and had the option to withdraw from the study at any time.

## Declaration of competing interest

The authors declare that they have no known competing financial interests or personal relationships that could have appeared to influence the work reported in this paper.

## Data Availability

The data that has been used is confidential.

## Appendix A Supplementary data

Supplementary material: **Table S1** The differences in the physicochemical properties of *Xiaoqu* inoculated with different combinations of molds. **Table S2** The volatile aroma compounds identified and quantified in *Zaopei* samples. **Table S3** VIP values of F1 and CK(J) in the fermentation process of *Baijiu*. **Table S4** The volatile aroma compounds identified and quantified in *Baijiu* samples Image 1
