# Supplementary material for: Study on the preparation of compound mold enhanced Xiaoqu and its effect on the yield and flavor of Qingxiangxing baijiu
Source: Food Chem X. 2025 Jul 3;29:102721. doi: 10.1016/j.fochx.2025.102721 (PMC12272614; doi:10.1016/j.fochx.2025.102721)
Supplement: Supplementary file 1 — Supplementary material: Table S1 The differences in the physicochemical properties of Xiaoqu inoculated with different combinations of molds. Table S2 The volatile aroma compounds identified and quantified in Zaopei samples. Table S3 VIP values of F1 and CK(J) in the fermentation process of Baijiu. Table S4 The volatile aroma compounds identified and quantified in Baijiu samples [file mmc1.docx]

**Study on the preparation of compound mold enhanced *Xiaoqu* and its effect on the yield and flavor of *Qingxiangxing Baijiu***

Tongwei Guan^a,^ *, Xinrui Yin^a^, Yuhang Jiang^a^, Yu Li^b^, Yuan Rao^b^, Jiayan Shao^c^, Ying Liu^a^, Lei Tian^a^, Yicheng Mao^d^, Xia Wang^c^

1. College of Food and Biological Engineering, Xihua University; Food Microbiology Key Laboratory of Sichuan Province, Chengdu 610039, China
2. Sichuan Tujiu Liquor Co., Ltd, Nanchong 637919, China
3. Chongqing Jiangji Distillery Co., Ltd, Jiangjin 402260, Chongqing, China
4. Xinjiang kaiduhe Liquor Co., Ltd, Xinjiang Uygur Autonomous Region 830000, China

*Author for correspondence: Tongwei Guan

Tel/Fax: +86 028 87720552

E-mail: guantongweily@163.com

**Supplementary Materials**

**Table S1** The differences in the physicochemical properties of *Xiaoqu* inoculated with different combinations of molds

|  |  |  | Moisture (%) | pH | Acidity  (mmol/10g) | Saccharifying  power  （mg/g.h） | Fermenting  power  (g/0.5g.72h) | Esterifying  Power  (mg/50g.72h) | Starch content  (%) | Liquefaction power  (mg/g.h) | The amount of mold  (×10^8^个/g) |
| --- | --- | --- | --- | --- | --- | --- | --- | --- | --- | --- | --- |
| F ^a^ | F1 | 2:1 | 9.4±0.96 ns | 6.63±0.24 ns | 0.8±0.01 ns | 1256±89.11 ns | 3.78±0.31*** | 710±66.83* | 33.6±3.13 ns | 1405±58.01 ns | 56.31±2.37**** |
|  | F2 | 1:1 | 10.0±1.00 ns | 6.31±0.58 ns | 0.9±0.03 ns | 798±38.00*** | 3.31±0.33*** | 532±53.00*** | 36.1±2.21 ns | 751±30.07**** | 38.71±1.73 ns |
|  | F3 | 1:2 | 7.0±0.52* | 6.77±0.33 ns | 0.7±0.06 ns | 648±54.75*** | 2.21±0.09*** | 156±17.61*** | 26.9±4.03*** | 1309±44.79** | 54.40±2.01**** |
|  |  |  |  |  |  |  |  |  |  |  |  |
| Q ^a^ | Q1 | 2:1 | 10.0±1.00 ns | 6.09±0.56 ns | 1.1±0.09** | 1293±79.00 ns | 2.90±0.20*** | 546±66.66** | 34.5±2.11 ns | 1107±50.71**** | 54.07±3.66**** |
|  | Q2 | 1:1 | 8.9±0.90 ns | 5.82±0.15 ns | 1.4±0.04*** | 940±48.15*** | 3.94±0.21*** | 159±15.00*** | 33.6±3.13 ns | 660±29.79**** | 35.97±2.56 ns |
|  | Q3 | 1:2 | 15.5±0.41*** | 6.45±0.45 ns | 0.5±0.02** | 833±41.36*** | 1.79±0.10*** | 145±13.89*** | 23.8±1.62*** | 675±41.07**** | 52.97±2.77**** |
|  |  |  |  |  |  |  |  |  |  |  |  |
| U1 | | | 8.9±0.90 ns | 5.71±0.50* | 0.9±0.04 ns | 679±40.50*** | 3.01±0.03 ns | 520±41.74*** | 33.6±3.13 ns | 890±35.79**** | 34.09±2.17 ns |
| W2 | | | 10.0±1.50 ns | 5.08±0.38*** | 1.7±0.26*** | 456±42.00*** | 1.68±0.02*** | 159±15.00*** | 21.2±1.99*** | 351±17.64**** | 35.31±2.11 ns |
| W3 | | | 15.0±0.95*** | 6.79±1.22 ns | 0.5±0.25** | 964±47.10*** | 1.52±0.11*** | 141±12.19*** | 21.5±1.66*** | 889±40.17**** | 37.08±1.85 ns |
| CK(J)^b^ | | | 9.9±1.55 | 6.58±0.32 | 0.8±0.08 | 1242±98.00 | 6.43±0.76 | 639±60.00 | 35.9±3.10 | 1421±67.71 | 35.74±1.89 |
| CK^c^ | | | 9.1±0.99 ns | 6.68±0.11 ns | 0.7±0.06 ns | 1140±89.11 ns | 3.07±0.308*** | 264±13.00*** | 25.7±2.56*** | 1259±44.09**** | 36.99±2.01 ns |
| Huaxi | | | 11.0±4.5 ns | 6.44±0.42 ns | 0.5±0.03** | 576±35.00*** | 5.63±0.10*** | 266±26.65*** | 30.8±1.54* | 1223±23.01**** | 34.56±1.79 ns |
| Anqi | | | 6.0±0.44*** | 6.37±0.50 ns | 0.4±0.04*** | 1176±100.60 ns | 2.27±0.12*** | 505±41.00*** | 30.1±2.89** | 1109±45.07**** | 36.07±2.56 ns |

^a^ "F" represents the proportion of U1 to W3, while "Q" represents the proportion of W2 to W3.

CK(J): Control *Xiaoqu* with mixed yeast produced by the distillery's fermentation process;

CK: Control *Xiaoqu* without mixed yeast fermentation from the distillery;

Huaxi: Huaxi *Xiaoqu*;

Anqi: Anqi *Xiaoqu*.

*Represents P＜0.05; **represents P＜0.01; ***represents P＜0.001; ****represents P＜0.0001; ns represents P＞0.0

**Table S2** The volatile aroma compounds identified and quantified in *Zaopei* samples

| Compounds Name (185) | CAS | Concentration (ug/kg) | | | | | |
| --- | --- | --- | --- | --- | --- | --- | --- |
|  |  | CK(J)-1 | CK(J)-4 | CK(J)-7 | F1-1 | F1-4 | F1-7 |
| **Alcohols (39)** |  |  |  |  |  |  |  |
| Ethanol | [64-17-5] | 453.43±33.37 | 1945.10±67.56 | 2194.57±67.44 | 915.23±55.34 | 6337.70±93.53 | 6825.07±95.43 |
| 2-Octanol | [123-96-6] | 8187.25±106.58 | 8187.25±106.58 | 8187.25±106.58 | 8187.25±106.58 | 8187.25±106.58 | 8187.25±106.58 |
| 1-Pentanol | [71-41-0] | 1194.80±67.37 | 3488.86±78.36 | 4044.31±80.36 | 937.81±47.12 | 2867.84±66.69 | 3602.49±80.48 |
| 1-Decanol | [112-30-1] | nd | nd | nd | nd | nd | 16.67±1.45 |
| 1-Eicosanol | [629-96-9] | 204.24±18.65 | nd | nd | nd | nd | nd |
| 2,3-Butanediol | [513-85-9] | 82.45±7.34 | 227.16±15.45 | 223.80±14.48 | 118.53±10.07 | 1607.12±70.56 | 740.39±44.76 |
| Dimethylsilanediol | [1066-42-8] | 847.42±52.77 | 424.26±32.12 | 708.44±48.77 | 464.16±39.32 | 1848.24±73.67 | 551.48±32.86 |
| 1-Octanol | [111-87-5] | 11.63±1.22 | nd | nd | 16.22±1.45 | nd | nd |
| Levomenthol | [2216-51-5] | 20.41±2.32 | nd | nd | nd | nd | nd |
| L-α-Terpineol | [10482-56-1] | 9.38±0.99 | nd | nd | nd | nd | nd |
| Heptaethylene glycol | [5617-32-3] | 178.44±15.16 | 821.06±45.67. | nd | nd | nd | 118.35±10.08 |
| Phenylethyl alcohol | [60-12-8] | 354.71±31.34 | 177.71±14.37 | 2658.57±69.07 | 340.35±26.09 | 4059.41±83.57 | 3413.30±78.98 |
| 1-lsopropoxypropan-2-ol | [3944-36-3] | Nd | 295.74±23.64 | nd | nd | nd | nd |
| 2-Methyl-1-propanol | [78-83-1] | nd | 41.90±3.87 | 587.82±33.43 | 127.80±10.03 | 320.93±25.04 | nd |
| 3-Methyl-3-buten-1-ol | [763-32-6] | nd | 38.35±3.45 | nd | 19.34 | nd | nd |
| 6-Methylheptanol | [1653-40-3] | nd | 43.83±4.08 | nd | nd | nd | nd |
| Isopropyl alcohol | [67-63-0] | nd | 29.61±2.43 | nd | nd | 66.83±6.22 | nd |
| Mercaptamine | [60-23-1] | nd | 59.12±5.66 | 46.59±4.36 | nd | nd | nd |
| 1-Nonanol | [143-08-8] | nd | 37.64±3.24 | nd | nd | 64.24±6.11 | 49.30±4.39 |
| 2-Nonanol | [628-99-9] | nd | nd | 109.87±9.78 | 39.89±3.78 | nd | nd |
| 3-Methylthiopropanol | [505-10-2] | nd | 61.86±5.77 | 45.07±4.30 | nd | 117.92±10.12 | 82.58±7.98 |
| 1-Undecanol | [112-42-5] | nd | 35.68±3.23 | nd | nd | 63.50±6.11 | nd |
| 2,5-Dimethyl-2,5-hexanediol | [110-03-2] | nd | 50.21±4.54 | 28.62±2.36 | 10.45±0.99 | 62.04±5.89 | 21.75±1.89 |
| Benzyl alcohol | [100-51-6] | nd | 76.29±6.68 | 58.72±5.48 | 9.85±0.92 | 88.11±8.35 | 67.29±6.23 |
| 3-Methyl-1-Butanol | [123-51-3] | nd | nd | 62.61±5.80 | nd | nd | nd |
| 1-Hexanol | [111-27-3] | nd | nd | 41.89±3.97 | nd | nd | 32.82 |
| 2-Furanmethanol | [98-00-0] | nd | nd | 24.93±2.15 | nd | 81.75±8.02 | 71.62±6.90 |
| 1,2-Hexanediol | [6920-22-5] | nd | nd | 12.68±1.09 | nd | nd | 24.97±2.17 |
| Hexaethylene glycol | [2615-15-8] | nd | nd | 54.40±4.93 | 0.81±0.01 | 56.71±5.15 | 31.15±2.78 |
| 3-Methoxy-1,2-propanediol | [623-39-2] | nd | nd | nd | 41.16±3.88 | nd | nd |
| 3-Methyl-1-butanol | [123-51-3] | nd | nd | nd | nd | nd | 21.62±1.94 |
| 1,3-Dichloro-2-propanol | [96-23-1] | nd | nd | nd | 13.46±1.09 | nd | nd |
| Behenic alcohol | [661-19-8] | nd | nd | nd | 71.04±6.77 | 145.98±12.25 | nd |
| 2-Tetradecanol | [4706-81-4] | nd | nd | nd | nd | 132.20±11.77 | nd |
| 2-Heptanol | [543-49-7] | nd | nd | nd | nd | 18.30±1.45 | nd |
| 1-Eicosanol | [629-96-9] | nd | nd | nd | nd | 30.48±2.78 | nd |
| 3-Methyl-2-hexanol | [2313-65-7] | nd | nd | nd | nd | nd | 8.58±0.67 |
| 5-Methyl-2-hexanol | [627-59-8] | nd | nd | nd | nd | nd | 18.53±1.45 |
| Diisobutylcarbinol | [108-82-7] | nd | nd | nd | nd | nd | 19.86±1.67 |
| 3,7,11-Trimethyl-1-dodecanol | [6750-34-1] | nd | nd | nd | nd | nd | 52.63±5.11 |
| **Esters (59)** |  |  |  |  |  |  |  |
| Ethyl acetate | [141-78-6] | 7.03±0.66 | 2590.94±67.88 | 4304.55±88.46 | 2.48±0.21 | 4100.29±85.48 | 4507.16±89.98 |
| Ethyl lactate | [97-64-3] | 21.26±2.14 | 747.01±43.35 | 648.49±39.09 | nd | nd | nd |
| Ethyl hexanoate | [123-66-0] | 23.83±2.15 | 3638.21±73.30 | 721.66±41.22 | 55.42±4.76 | 822.64±46.78 | 766.95±44.12 |
| Caproic acid propyl ester | [626-77-7] | nd | nd | nd | nd | nd | 18.00±1.34 |
| Vinyl formate | [692-45-5] | 45.93±3.93 | nd | nd | nd | nd | nd |
| n-Propyl acetate | [109-60-4] | 211.43±18.55 | nd | nd | 336.44±26.34 | nd | nd |
| Isobutyl acetate | [110-19-0] | 172.96±10.25 | 66.03±6.23 | 68.66±6.17 | 96.59±8.75 | 59.97±4.98 | 86.90±7.83 |
| lsopentyl acetate | [123-92-2] | 1038.90±56.56 | nd | 4573.89±80.56 | 654.81±43.22 | 1065.19±57.82 | 1717.60±60.77 |
| 2-Phenylethyl hexanoate | [6290-37-5] | 0.81±0.02 | nd | nd | nd | nd | 7.27±0.68 |
| Benzyl acetate | [140-11-4] | 20.75±2.19 | nd | nd | nd | nd | nd |
| Phenethyl acetate | [103-45-7] | 78.98±6.28 | nd | 1054.25±56.66 | 108.77±9.45 | nd | 1162.24±58.34 |
| (E)-9-Octadecenoic acid ethyl ester | [6114-18-7] | 16.34±1.29 | nd | nd | nd | nd | nd |
| Ethyl myristate | [124-06-1] | 73.56±7.10 | 219.62±15.44 | 2871.30±68.99 | 114.47±10.05 | 2871.54±69.09 | 2880.90±69.46 |
| Butyl octyl phthalate | [84-78-6] | 8.70±0.75 | nd | nd | 4.89±0.38 | nd | 4.21±0.36 |
| Diisobutyl phthalate | [84-69-5] | nd | nd | nd | 17.95±1.45 | 24.37±2.16 | 32.30±2.67 |
| Ethyl oleate | [111-62-6] | 17.42±1.44 | 805.25±45.09 | 550.43±33.78 | 41.02±3.22 | 603.79±35.24 | 255.24±23.88 |
| Linoleic acid ethyl ester | [544-35-4] | 9.35±0.87 | 1049.66±56.78 | 530.15±32.55 | 24.70±1.98 | 40.49±3.78 | 306.96±25.25 |
| Ethyl butyrate | [105-54-4] | nd | 150.34±13.08 | 131.75±11.07 | nd | 100.24±0.95 | 82.43±7.44 |
| n-Pentyl propionate | [624-54-4] | nd | 94.17±9.04 | nd | nd | nd | nd |
| Ethyl heptanoate | [106-30-9] | nd | 69.51±6.50 | 49.77±4.47 | nd | 48.32±4.42 | 60.17±5.34 |
| sec-Butyl nitrite | [924-43-6] | nd | 30.26±2.87 | 4.97±0.46 | nd | nd | 105.61±0.96 |
| Butyl hexanoate | [626-82-4] | nd | 16.93±1.44 | 8.30±0.79 | nd | 27.38±2.54 | 4.78±0.36 |
| Ethyl caprylate | [106-32-1] | nd | 56.23±5.25 | 2061.51±65.64 | 37.66±3.34 | 2542.87±68.32 | 2321.74±66.12 |
| Isopentyl hexanoate | [2198-61-0] | nd | 32.77±2.99 | nd | nd | 34.13±3.10 | 16.39±1.45 |
| Propyl octanoate | [624-13-5] | nd | 71.57±6.94 | nd | nd | nd | nd |
| Etheyl octanoat | [2306-88-9] | nd | nd | nd | nd | 55.61±5.14 | 56.51±5.22 |
| Butyrolactone | [96-48-0] | nd | 24.77±2.09 | 16.37±1.42 | nd | 35.52±3.24 | 12.77±1.11 |
| Ethyl benzoate | [93-89-0] | nd | 3366.58±73.98 | nd | nd | nd | nd |
| Diethyl succinate | [123-25-1] | nd | 3104.65±72.67 | 96.02±9.12 | 48.74±4.25 | 1305.44±57.22 | 1557.46±59.54 |
| Ethyl undecanoate | [627-90-7] | nd | 42.30±3.78 | 28.48±2.16 | nd | nd | 14.24±1.19 |
| Ethyl laurate | [106-33-2] | nd | nd | 719.19±43.76 | nd | 798.27±44.46 | 753.31±43.46 |
| Palmitic acid propyl ester | [2239-78-3] | nd | 15.81±1.37 | 7.39±0.66 | nd | 23.95±2.11 | 15.81±1.25 |
| n-Capric acid isoamyl ester | [2306-91-4] | nd | 41.31±37.59 | 24.10±2.13 | nd | 34.04±3.23 | 33.64±3.01 |
| Ethyl (E)-cinnamate | [4192-77-2] | nd | 27.38±2.43 | nd | nd | nd | nd |
| Isoamyl propionate | [105-68-0] | nd | nd | 99.66±9.67 | nd | nd | nd |
| n-Pentyl propionate | [624-54-4] | nd | nd | nd | nd | 56.01±4.76 | nd |
| Amyl butyrate | [540-18-1] | nd | nd | 4.74±0.41 | nd | 16.30±1.23 | 17.91±1.51 |
| Ethyl nonanoate | [123-29-5] | nd | nd | 59.82±5.65 | nd | 45.67±4.20 | 41.59±3.78 |
| Trimethylene acetate | [628-66-0] | nd | nd | 131.90±10.22 | nd | nd | 197.89±14.98 |
| Ethyl caprate | [110-38-3] | nd | nd | 2122.44±67.22 | nd | 2661.50±67.45 | nd |
| lsoamyl caprylate | [2035-99-6] | nd | nd | 45.62±4.21 | nd | nd | nd |
| Ethyl phenylacetate | [101-97-3] | nd | nd | 70.63±6.65 | nd | nd | 130.22±1.11 |
| n-Propyl decanoate | [30673-60-0] | nd | nd | 11.75±1.09 | nd | nd | nd |
| Ethyl 3-phenylpropionate | [2021-28-5] | nd | nd | 60.93±5.33 | nd | 84.45±7.98 | 74.29±6.98 |
| Ethyl heptadecanoate | [14010-23-2] | nd | nd | 14.04±1.21 | nd | nd | nd |
| Methyl carbamate | [598-55-0] | nd | nd | 4.70±0.34 | nd | nd | nd |
| Allyl 2-ethyl butyrate | [7493-69-8] | nd | nd | 8.98±0.78 | nd | nd | nd |
| Hexyl hexanoate | [6378-65-0] | nd | nd | 113.74±10.23 | nd | 486.82±46.56 | nd |
| Ethyl valerate | [539-82-2] | nd | nd | nd | nd | 31.55±2.91 | 31.58±0.28 |
| Caproic acid propyl ester | [626-77-7] | nd | nd | nd | nd | 23.56±2.11 | nd |
| Isosorbide Dinitrate | [87-33-2] | nd | nd | nd | nd | 13.00±1.12 | nd |
| Acetic acid octyl ester | [112-14-1] | nd | nd | nd | nd | 35.85±2.98 | 5.14±0.44 |
| Acetic acid nonyl ester | [143-13-5] | nd | nd | nd | nd | nd | 27.75±2.45 |
| Propyl octanoate | [624-13-5] | nd | nd | nd | nd | 100.04±9.18 | nd |
| Styralyl acetate | [93-92-5] | nd | nd | nd | nd | 7.76±0.67 | nd |
| Ethyl 3-hydroxyoctanoate | [7367-90-0] | nd | nd | nd | nd | 14.80±1.23 | 6.20±0.54 |
| Ethyl linoleate | [7619-08-1] | nd | nd | nd | nd | 581.94±34.34 | 256.25±23.31 |
| gamma-Nonanolactone | [104-61-0] | nd | nd | nd | nd | 53.46±4.89 | 41.66±3.22 |
| Diethylene glycol monolaurate | [141-20-8] | nd | nd | nd | nd | 17.10±1.45 | nd |
| **Acids (27)** |  |  |  |  |  |  |  |
| Formic acid | [64-18-6] | nd | 13734.12±123.94 | nd | nd | nd | nd |
| Acetic acid | [64-19-7] | 236.21±19.23 | nd | 3132.27±73.34 | 388.25±25.78 | 22.88±1.76 | 3363.66±78.23 |
| Butanoic acid | [107-92-6] | nd | nd | nd | nd | 163.66±1.45 | 148.64±13.45 |
| Isovaleric acid | [503-74-2] | nd | nd | 68.15±6.56 | nd | nd | 103.84±9.44 |
| DL-3-Methylvaleric acid | [105-43-1] | nd | nd | nd | nd | 11.78±1.01 | 47.70±4.13 |
| Isobutyric acid | [79-31-2] | 27.44±2.51 | 89.93±8.42 | 52.52±4.87 | 26.06±2.33 | nd | 37.79±3.23 |
| 4-Methylvaleric acid | [646-07-1] | 12.80±1.13 | nd | nd | 11.63±1.09 | nd | nd |
| 2-Hydroxymyristic acid | [2507-55-3] | 7.71±0.79 | nd | nd | nd | nd | nd |
| Erucic acid | [112-86-7] | 38.93±3.14 | nd | nd | 8.64±0.76 | nd | nd |
| 3-(3-Carboxy-4-hydroxyphenyl)-D-alanine | [4303-95-1] | nd | nd | nd | nd | nd | nd |
| Oxalic acid | [144-62-7] | nd | 622.52±37.73 | nd | nd | 1052.55±55.12 | nd |
| Propanoic acid | [79-09-4] | nd | 258.29±19.46 | 174.80±14.33 | 12.39±1.07 | 317.15±25.35 | 229.77±16.34 |
| Hexanoic acid | [142-62-1] | nd | 353.15±26.23 | 73.60±6.83 | 61.39±5.67 | nd | 294.17±24.56 |
| Octanoic acid | [124-07-2] | nd | 143.41±12.34 | 67.19±6.22 | nd | 538.83±34.11 | 227.96±16.42 |
| Pentanoic acid | [109-52-4] | nd | nd | nd | nd | nd | 12.76±1.01 |
| n-Decanoic acid | [334-48-5] | nd | 33.17±3.11 | 23.45±1.92 | nd | 128.32±10.07 | 51.31±4.78 |
| 3-Nitropropanoic acid | [504-88-1] | nd | 49.17±4.66 | nd | 34.27±2.75 | nd | nd |
| trans-13-Octadecenoic acid | [693-71-0] | nd | nd | nd | 13.36±1.12 | 86.06±7.88 | nd |
| Oleic Acid | [112-80-1] | nd | nd | nd | nd | 6.81±0.58 | nd |
| 3-Methyloxirane-2-carboxylic acid | [2443-40-5] | nd | nd | nd | nd | 9.17±0.86 | nd |
| 3-(3-Carboxy-4-hydroxyphenyl)-D-alanine | [4303-95-1] | nd | nd | nd | nd | 43.96±4.11 | 15.21±1.24 |
| Heptanoic acid | [111-14-8] | nd | nd | nd | nd | 33.32±3.10 | 10.70±0.98 |
| Nonanoic acid | [112-05-0] | nd | nd | nd | nd | 30.08±2.67 | nd |
| 4-Amino-1,5-pentandioic acid | [7766-85-0] | nd | nd | nd | nd | 24.15±2.12 | nd |
| Glyceric acid | [473-81-4] | nd | nd | nd | nd | nd | 1.43±0.11 |
| Arginine | [74-79-3] | nd | nd | nd | nd | nd | 4.03±0.32 |
| **Ketones (13)** |  |  |  |  |  |  |  |
| 4-Hydroxy-2-butanone | [590-90-9] | 4716.55±86.75 | 330.41±31.37 | nd | nd | nd | nd |
| Acetoin | [513-86-0] | 27.75±2.29 | nd | 35.27±3.22 | 17.18±1.45 | 109.66±0.98 | nd |
| 2-Octanone | [111-13-7] | 2339.74±64.79 | 139.83±11.44 | 86.55±8.26 | 1090.46±56.43 | 70.34±6.54 | 122.21±10.23 |
| 2-Dodecanone | [6175-49-1] | 5.39±0.12 | nd | nd | nd | nd | nd |
| Fitone | [502-69-2] | 17.98±1.09 | nd | nd | 12.18±1.02 | nd | nd |
| Acetophenone | [98-86-2] | 23.17±2.18 | nd | nd | 28.03±2.45 | nd | nd |
| 3-Methyl-1-phenyl-2-butanone | [2893-05-2] | nd | 54.18±5.12 | nd | nd | 56.91±4.45 | nd |
| 3-Octanone | [106-68-3] | nd | nd | nd | nd | 7.62±0.61 | nd |
| 3-Amino-2-oxazolidinone | [80-65-9] | nd | nd | nd | nd | nd | 7.42±0.65 |
| 6-Methyl-5-hepten-2-one | [110-93-0] | nd | nd | nd | nd | nd | 4.72±0.43 |
| 2-Hydroxy-3-pentanone | [5704-20-1] | nd | nd | nd | nd | nd | 17.58±1.56 |
| 8-Hydroxy-2-octanone | [25368-54-1] | nd | nd | 18.58±1.65 | nd | nd | nd |
| Geranyl acetone | [3796-70-1] | nd | nd | 9.27±0.84 | nd | nd | nd |
| **Aldehydes (10)** |  |  |  |  |  |  |  |
| Decanal | [112-31-2] | 16.67±1.05 | 21.52±1.96 | 21.80±1.98 | 12.17±1.03 | 51.85±4.13 | 29.61±2.42 |
| Benzeneacetaldehyde | [122-78-1] | 26.35±2.18 | 224.98±2.09 | 149.48±13.45 | 17.57±1.34 | 340.24±26.13 | 192.68±14.32 |
| Cuminaldehyde | [122-03-2] | 27.21±2.28 | nd | nd | nd | nd | nd |
| p-Anisaldehyde | [123-11-5] | 28.51±2.13 | nd | nd | nd | nd | nd |
| Benzyl alcohol | [100-51-6] | 38.51±3.22 | nd | nd | nd | nd | nd |
| gamma-Nonanolactone | [104-61-0] | nd | 35.61±3.21 | 22.25±2.08 | nd | nd | nd |
| Nonanal | [124-19-6] | nd | nd | 91.43±8.22 | 14.51±1.23 | 22.38±1.98 | 52.82±4.89 |
| Aldol | [107-89-1] | nd | nd | nd | nd | 22.23±1.96 | nd |
| (E)-2-Octenal | [2548-87-0] | nd | nd | 26.60±2.13 | nd | nd | nd |
| 2-Carboxybenzaldehyde | [119-67-5] | nd | nd | nd | nd | nd | 2.97±0.25 |
| **Phenols (9)** |  |  |  |  |  |  |  |
| 2-methoxy-Phenol | [90-05-1] | 14.54±1.12 | 47.73±4.37 | 29.09±2.34 | 21.01±1.99 | 71.22±6.61 | 52.54±4.98 |
| Phenol | [108-95-2] | 6.20±0.56 | 21.48±1.88 | 14.20±1.24 | 6.96±0.59 | 31.81±2.78 | 48.18±4.34 |
| 4-Ethyl-2-methoxyphenol | [2785-89-9] | 6.15±0.54 | 165.21±13.12 | 95.17±8.97 | nd | 191.10±14.34 | 142.85±12.28 |
| 2-Ethylphenol | [90-00-6] | 4.62±0.43 | 25.72±2.16 | 14.77±1.33 | 10.48±0.99 | 32.48±2.93 | 12.70±1.09 |
| 2-Methoxy-4-vinylphenol | [7786-61-0] | 20.66±1.95 | 22.41±2.04 | 12.01±1.01 | 44.59±3.67 | 317.95±25.76 | 419.07±32.22 |
| 2,4-Di-tert-butylphenol | [96-76-4] | 29.81±2.20 | 43.45±3.93 | 20.36±1.72 | 28.01±2.55 | 56.52±4.87 | 21.68±2.01 |
| 4-Vinylphenol | [2628-17-3] | 9.69±0.91 | 32.75±3.09 | nd | nd | nd | nd |
| m-Cresol | [108-39-4] | nd | 48.12±4.35 | 30.77±2.67 | 3.21±0.28 | nd | nd |
| Phenol, 2,5-bis(1,1-dimethylethyl)- | [5875-45-6] | nd | nd | nd | nd | nd | 12.66±1.02 |
| **Hydrocarbons (21)** |  |  |  |  |  |  |  |
| Hexamethylcyclotrisiloxane | [541-05-9] | 164.31±15.78 | 239.02±22.36 | 353.63±26.34 | 64.21±5.98 | 1172.20±56.88 | 132.65±11.23 |
| Nitrosomethane | [865-40-7] | 1070.59±56.23 | 263.94±24.12 | nd | nd | nd | nd |
| Heptadecane | [629-78-7] | nd | nd | nd | nd | nd | 73.81±6.29 |
| Tridecane | [629-50-5] | 106.06±9.12 | nd | nd | 65.73±5.98 | 72.30±6.34 | nd |
| Nonadecane | [629-92-5] | 746.16±40.45 | nd | nd | 232.42±16.34 | 449.85±33.67 | 312.89±26.36 |
| Tetradecane | [629-59-4] | 250.60±24.33 | 129.14±11.12 | 98.44±9.06 | 7.74±0.67 | 344.26±27.89 | 213.32±17.78 |
| Octadecanal | [638-66-4] | 11.31±1.11 | nd | nd | nd | 8.22±0.76 | nd |
| 1,2-Epoxyoctadecane | [7390-81-0] | 29.50±2.81 | nd | nd | nd | nd | nd |
| Octamethylcyclotetrasiloxane | [556-67-2] | nd | 750.34±73.22 | 338.15±26.78 | 116.00±10.02 | 478.27±34.78 | 549.75±4.98 |
| 3-Methylundecane | [1002-43-3] | nd | 45.33±4.32 | 34.69±2.67 | nd | 34.22±3.22 | 22.65±2.02 |
| Dodecane | [112-40-3] | nd | 102.92±9.97 | 34.42±2.63 | nd | 65.16±5.22 | nd |
| Hexadecane | [544-76-3] | nd | 65.94±6.11 | nd | nd | 127.23±10.10 | 45.89±4.29 |
| 3-Methyltridecane | [6418-41-3] | nd | 108.91±10.55 | 28.22±2.45 | nd | nd | nd |
| Dodecamethylpentasiloxane | [141-63-9] | nd | 365.15±26.34 | nd | 221.28±16.43 | nd | 134.50±11.22 |
| Ethyl glycidyl ether | [4016-11-9] | nd | 42.19±3.96 | nd | nd | 15.71±1.22 | nd |
| Dimethylsilane | [1111-74-6] | nd | 21.52±2.09 | nd | nd | nd | nd |
| Pentadecane | [629-62-9] | nd | nd | nd | 76.49±6,78 | nd | nd |
| Tetradecamethylhexasiloxane | [107-52-8] | nd | nd | nd | 70.61±6.56 | nd | nd |
| 3,3,4,4-Tetrafluorohexane | [648-36-2] | nd | nd | 28.91±2.50 | nd | 77.12±6.98 | nd |
| 1,2-Dibutoxyethane | [112-48-1] | nd | nd | nd | nd | nd | 247.08±22.25 |
| Fluorotriethylsilane | [358-43-0] | nd | nd | 133.94±10.22 | nd | nd | nd |
| **Furans (4)** |  |  |  |  |  |  |  |
| Tetradecahydrocyclododeca[c]furan | [42824-62-4] | 133.42±12.15 | nd | nd | nd | nd | nd |
| 2,3-Dihydrobenzofuran | [496-16-2] | nd | nd | 21.05±1.99 | 11.68±1.02 | 61.85±5.78 | 35.15±3.12 |
| 2-Pentylfuran | [3777-69-3] | nd | nd | 19.09±1.67 | nd | nd | 8.70±0.77 |
| 2,2,4,4-Tetramethyltetrahydrofuran | [3358-28-9] | nd | nd | 3.66±0.30 | nd | nd | nd |
| **Sulfides (1)** |  |  |  |  |  |  |  |
| Diallyl disulphide | [2179-57-9] | 16.24±1.46 | 23.46±2.12 | 9.35±0.89 | nd | 14.95±1.02 | 21.51±1.78 |
| **Others (2)** |  |  |  |  |  |  |  |
| Anethole | [104-46-1] | 112.15±10.15 | nd | nd | 55.23±4.58 | nd | nd |
| L-Glucose | [921-60-8] | nd | nd | nd | nd | nd | 33.22±2.98 |

Note: CK(J)-1 represents the *Zaopei* of the control group CK(J) on the first day of fermentation; CK(J)-4 represents the *Zaopei* of the control group CK(J) on the 4th day of fermentation; CK(J)-7 represents the *Zaopei* of the control group CK(J) on the 7th day of fermentation; F1-1 represents the *Zaopei* of the first day of the fermentation of *Xiaoqu* F1 enhanced by composite fungi; F1-4 represents the *Zaopei* of the 4th day of fermentation of *Xiaoqu* F1 enhanced by composite fungi; F1-7 represents the fermented mash on the 7th day of the fermentation of Xiaoqu F1 enhanced by composite fungi.

**Table S3** VIP values of F1 and CK(J) in the fermentation process of *Baijiu*

| Compounds Name | VIP |
| --- | --- |
| Ethyl acetate | 2.1524 |
| Acetic acid | 1.2018 |
| 2,3-Butanediol | 0.8164 |
| 2,4-Di-tert-butylphenol | 0.0013 |
| Ethyl lactate | 0.6989 |
| Phenylethyl alcohol | 0.4150 |
| 1-Hexanol | 0.0143 |
| Nonanal | 0.0316 |
| Ethyl hexanoate | 1.0468 |
| Phenethyl alcohol | 2.2933 |
| Decanal | 0.0138 |
| Benzeneacetaldehyde | 0.0945 |
| Geranyl acetone | 0.0054 |
| Ethyl caprate | 0.8727 |
| 2-Octanone | 0.9603 |
| lsopentyl acetate | 2.1182 |
| Hexanoic acid | 0.0528 |
| 2-methoxy-Phenol | 0.0272 |
| Ethyl nonanoate | 0.0238 |
| Octanoic acid | 0.2348 |

**Table S4** The volatile aroma compounds identified and quantified in *Baijiu* samples

| Compounds Name (45) | CAS | Concentration (mg/L) | |
| --- | --- | --- | --- |
|  |  | D | S |
| **Alcohols (15)** |  |  |  |
| Ethanol | [64-17-5] | 214.25±20.09 | 233.39±22.05 |
| Methanethiol | [74-93-1] | 274.70±25.56 | 130.27±12.22 |
| 1-Propoxy-2-propanol | [1569-01-3] | ND | 1.38±0.11 |
| 1-Propanol | [71-23-8] | ND | 19.70±1.82 |
| 2-Butanol | [78-92-2] | 32.66±2.99 | 3.09±0.29 |
| 2-Methyl-1-propanol | [78-83-1] | 57.05±5.34 | 53.52±5.11 |
| Isoamyl alcohol | [123-51-3] | 0.83±0.01 | ND |
| Isopropyl Alcohol | [67-63-0] | 1.76±0.15 | ND |
| 1-Butanol | [71-36-3] | 2.67±0.24 | 0.87±0.02 |
| 1-Pentanol | [71-41-0] | 153.81±13.11 | 116.84±10.33 |
| 2-Octanol | [123-96-6] | 5.09±0.45 | 1.91±0.16 |
| 2,3-Butanediol | [513-85-9] | 13.51±1.21 | 16.03±1.45 |
| Dimethylsilanediol | [1066-42-8] | 14.39±1.24 | 7.35±0.69 |
| Phenylethyl Alcohol | [60-12-8] | 8.43±0.69 | 1.30±0.11 |
| Hexaethylene glycol | [2615-15-8] | 7.82±0.71 | ND |
| **Esters (12)** |  |  |  |
| Ethyl Acetate | [141-78-6] | 192.42±16.89 | 225.25±20.02 |
| Ethyl butyrate | [105-54-4] | 6.71±0.56 | 1.74±0.14 |
| lsopentyl acetate | [123-92-2] | 52.31±4.98 | 39.73±3.78 |
| Ethyl hexanoate | [123-66-0] | 8.45±0.79 | 7.27±0.67 |
| Ethyl lactate | [97-64-3] | 40.67±3.87 | 26.39±2.34 |
| Etheyl octanoat | [106-32-1] | 43.23±4.11 | 44.09±4.23 |
| Diethyl succinate | [123-25-1] | 8.03±0.77 | 3.48±0.25 |
| Ethyl caprate | [110-38-3] | 33.19±3.11 | 28.26±2.56 |
| Phenylethyl acetate | [103-45-7] | 8.57±0.79 | 6.17±0.56 |
| Ethyl laurate | [106-33-2] | 21.22±1.98 | 7.18±0.67 |
| Ethyl myristate | [124-06-1] | ND | 13.34±1.22 |
| Ethyl Oleate | [111-62-6] | 1.97±0.18 | 2.89±0.25 |
| **Acids (2)** |  |  |  |
| Acetic acid | [64-19-7] | 70.64±6.87 | 88.27±8.56 |
| Propanoic acid | [79-09-4] | 2.31±0.22 | 2.86±0.24 |
| **Ketones (1)** |  |  |  |
| 2-Octanone | [111-13-7] | 9.53±0.93 | 11.50±1.02 |
| **Aldehydes (3)** |  |  |  |
| Acetaldehyde | [75-07-0] | 2.92±0.72 | 0.83±0.02 |
| Benzeneacetaldehyde | [122-78-1] | 2.66±0.25 | 2.90±0.25 |
| Acetal | [105-57-7] | 440.28±41.22 | 428.11±37.22 |
| **Phenols (1)** |  |  |  |
| Phenol | [108-95-2] | 0.82±0.02 | 0.26±0.02 |
| **Hydrocarbons (10)** |  |  |  |
| Hexamethylcyclotrisiloxane | [541-05-9] | 10.60±1.01 | 3.68±0.33 |
| Octamethyl cyclotetrasiloxane | [556-67-2] | 16.89±1.23 | 17.20±1.56 |
| 1,1-diethoxy-3-methyl-Butane | [3842-03-3] | 2.82±0.22 | 16.98±1.45 |
| 1,1-diethoxy-2-methyl | [3658-94-4] | ND | 1.56±0.15 |
| Dodecane | [112-40-3] | 2.30±0.20 | 1.85±0.16 |
| Tridecane | [629-50-5] | 29.93±2.22 | ND |
| Tetradecane | [629-59-4] | 3.21±0.27 | 4.86±0.43 |
| Hexadecane | [544-76-3] | ND | 1.84±0.16 |
| Nonadecane | [629-92-5] | ND | 0.87±0.02 |
| Cyclopentadecane | [295-48-7] | 0.32±0.01 | ND |
| **Others (1)** |  |  |  |
| 2-Pentylfunan | [3777-69-3] | ND | 0.92±0.03 |

Note: D represents the control group CK(J) original wine; S represents compound fungus enhanced *Xiaoqu* F1 original wine.


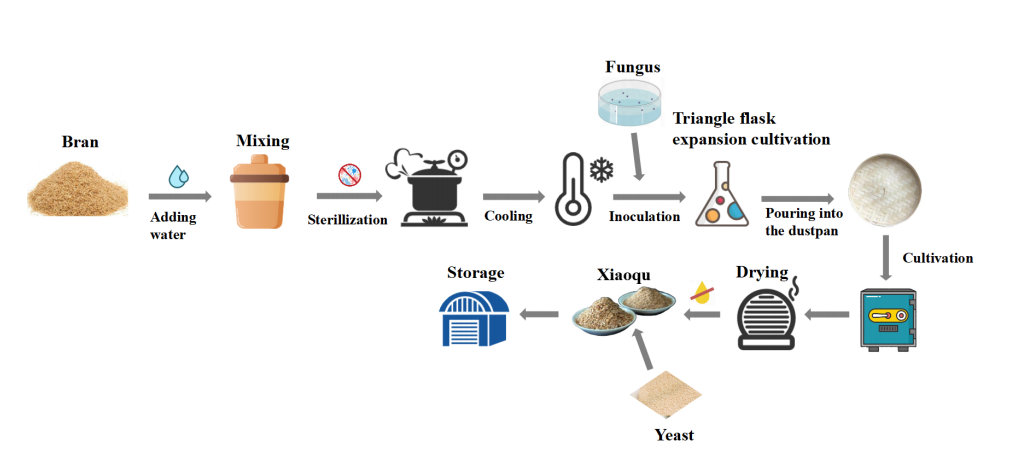


Figure. S1. Flowchart of Xiaoqu production process


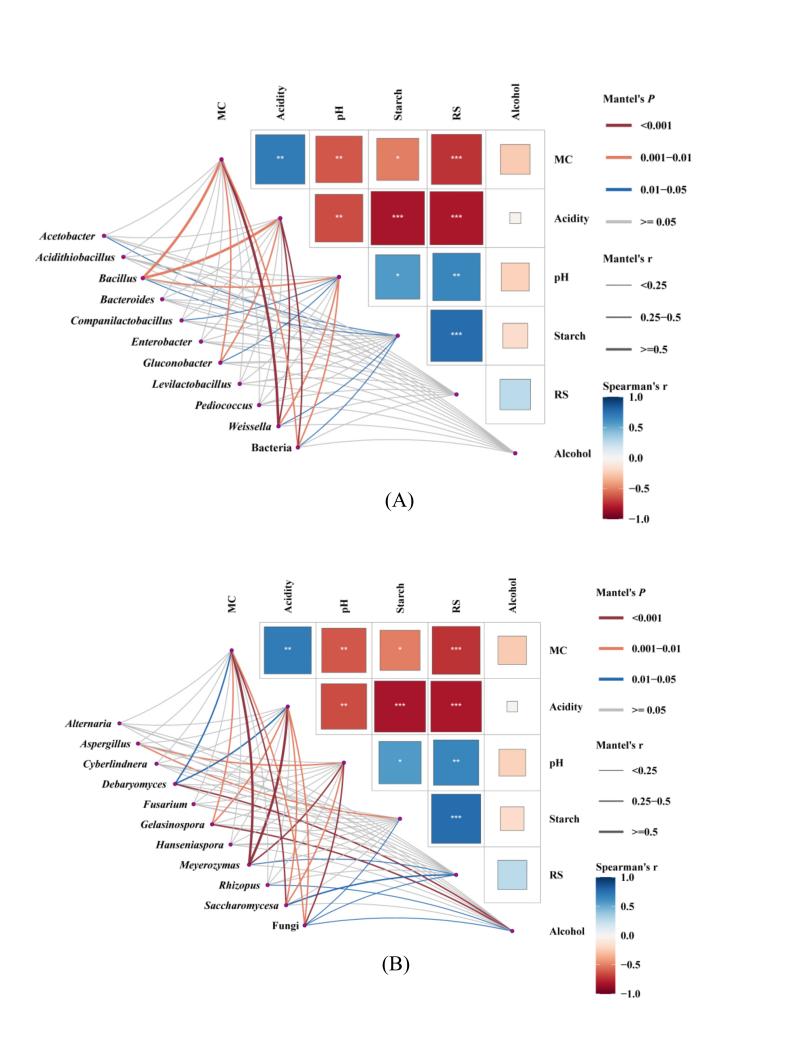


Figure S2. Correlation between physicochemical factors and microbial communities as well as physicochemical factors, (A) bacteria; (B) Fungi. The color and width of the lines correspond to the P and r values obtained through the Mantel test, respectively, and the color of the lines represents the positive and negative correlation of the Spearman test. The heat map shows the Spearman correlation between each physicochemical factor.


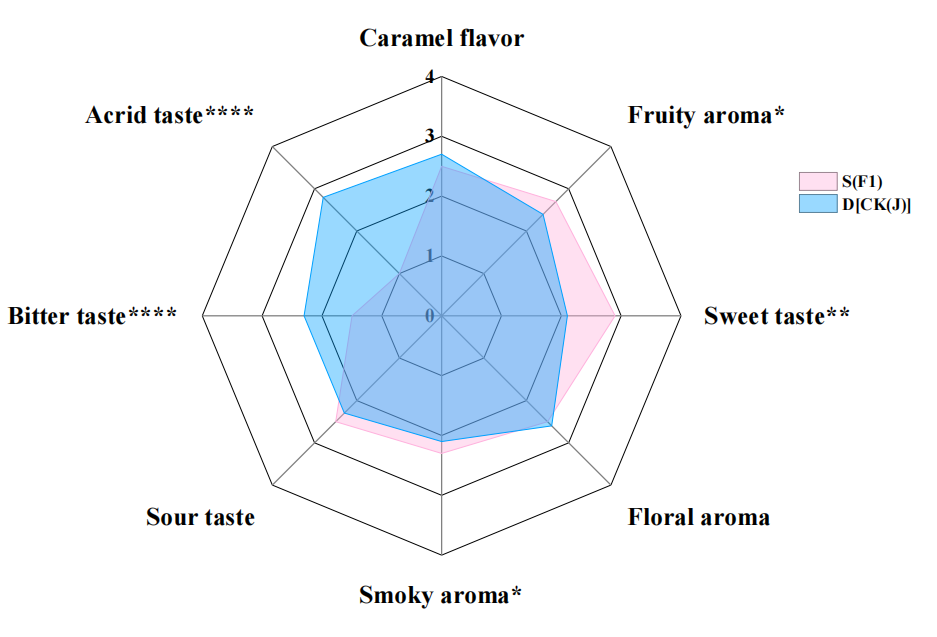


Figure S3.Quantitative descriptive analysis on the sensory profile of the distillate. Values are the mean of 10 assessors and three replicates. CK(J): Control *Xiaoqu* with mixed yeast produced by the distillery's fermentation process. F1: Inoculate fortified *Xiaoqu* with a mold combination of U1: W3=2:1.
